# Regulation of PPAR**α** by APP in Alzheimer disease affects the pharmacological modulation of synaptic activity

**DOI:** 10.1172/jci.insight.150099

**Published:** 2021-08-23

**Authors:** Francisco Sáez-Orellana, Thomas Leroy, Floriane Ribeiro, Anna Kreis, Karelle Leroy, Fanny Lalloyer, Eric Baugé, Bart Staels, Charles Duyckaerts, Jean-Pierre Brion, Philippe Gailly, Jean-Noël Octave, Nathalie Pierrot

**Affiliations:** 1 Alzheimer Dementia Group and; 2 Laboratory of Cell Physiology, Institute of Neuroscience, Catholic University of Louvain, Brussels, Belgium.; 3 Laboratory of Histology and Neuropathology, Free University of Brussels, Brussels, Belgium.; 4 University of Lille, INSERM, CHU Lille, Pasteur Institute of Lille, U1011, Lille, France.; 5 University of Sorbonne, Pitié-Salpêtrière University Hospital, and Paris Brain Institute, CNRS UMR7225, INSERM U1127, Paris, France.

**Keywords:** Neuroscience, Alzheimer disease, Mouse models

## Abstract

Among genetic susceptibility loci associated with late-onset Alzheimer disease (LOAD), genetic polymorphisms identified in genes encoding lipid carriers led to the hypothesis that a disruption of lipid metabolism could promote disease progression. We previously reported that amyloid precursor protein (APP) involved in Alzheimer disease (AD) physiopathology impairs lipid synthesis needed for cortical networks’ activity and that activation of peroxisome proliferator–activated receptor α (PPARα), a metabolic regulator involved in lipid metabolism, improves synaptic plasticity in an AD mouse model. These observations led us to investigate a possible correlation between PPARα function and full-length APP expression. Here, we report that PPARα expression and activation were inversely related to APP expression both in LOAD brains and in early-onset AD cases with a duplication of the *APP* gene, but not in control human brains. Moreover, human APP expression decreased *PPARA* expression and its related target genes in transgenic mice and in cultured cortical cells, while opposite results were observed in APP-silenced cortical networks. In cultured neurons, APP-mediated decrease or increase in synaptic activity was corrected by a PPARα-specific agonist and antagonist, respectively. APP-mediated control of synaptic activity was abolished following PPARα deficiency, indicating a key function of PPARα in this process.

## Graphical abstract

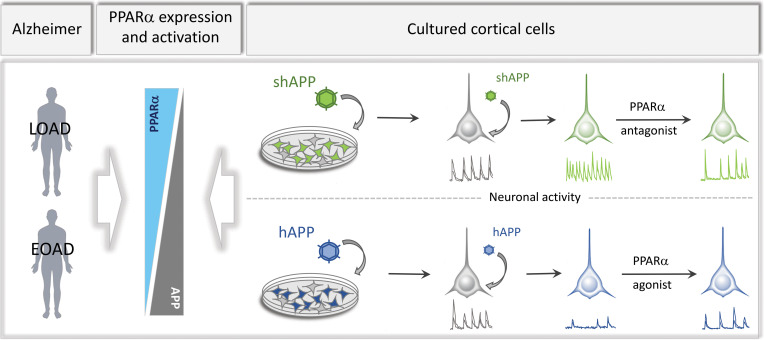

## Introduction

Alzheimer disease (AD) is the most common form of dementia, accounting for nearly 70% of the cases worldwide (1). AD is characterized by the presence in the brain of neurofibrillary tangles containing paired helical filaments of hyperphosphorylated tau protein and senile plaques, with an amyloid core of amyloid β (Aβ) peptide derived from the amyloid precursor protein (APP) (2). Synaptic dysfunction seems to occur long before the presence of these neuropathological lesions in the brains of AD patients and might contribute to cognitive dysfunction (3). Autosomal dominant mutations found in *APP* gene or genes encoding presenilins, involved in the γ-secretase–mediated processing of APP into Aβ, account for the majority of rare inherited early-onset AD (EOAD) cases, in which Aβ is considered the main culprit of the pathology (1). However, genome-wide association studies have identified dozens of genetic susceptibility loci that are associated with higher risk for late-onset AD (LOAD) (4). Among the most important AD genetic risk factors, genetic polymorphisms found first in *APOE* and later in *CLU* and *ABCA7* genes encoding lipid carriers (4) led to the hypothesis that a disruption of lipid metabolism could promote disease progression (5). This hypothesis is sustained by findings reporting that genetic polymorphisms in *SREBF* and *PPARA* genes, involved in cholesterol and fatty acid (FA) metabolism, were associated with an increased risk of LOAD (6–8), although the association between the genetic polymorphism identified in *PPARA* encoding the peroxisome proliferator–activated receptor α (PPARα) and AD is controversial (9). However, several LOAD genetic risk factors are involved in pathways that are governed by PPARα, highlighting a potential link between PPARα and the etiology of AD (reviewed in ref. 10). PPARα belongs to the nuclear receptor superfamily of ligand-dependent transcription factors and is broadly implicated in a wide variety of biological processes regulating energy balance, inflammation, and FA and glucose metabolism (11), a set of pathways previously reported to be disturbed in LOAD also (12–14). More recently, a potential role of PPARα in cognition emerged. Spatial learning and long-term memory deficits observed in PPARα-knockout mice indicate that PPARα is required for normal cognitive function (15). Moreover, PPARα deficiency affects the expression of a set of synaptic proteins involved in excitatory neurotransmission (16) and severely impairs hippocampal long-term potentiation (LTP) (17), an activity-dependent enhancement of synaptic strength involved in memory processing (18). Accordingly, a growing body of evidence reports that activation of PPARs has salutary effects on neurodegenerative disorders, including AD (19). Administration of PPARα activators reduces AD-like pathology and cognitive decline in murine models of AD overexpressing mutated human APP and presenilin 1 linked to familial AD (17, 20, 21). Inasmuch as PPARs’ expression is modified in AD brains (22), we hypothesized that the function of PPARα could be impaired in AD and may therefore contribute to the progression of the disease.

In this study, we report that *PPARA* mRNA level and the expression of PPARα-related target genes were modified in brains from LOAD and EOAD cases with a rare *APP* duplication and that *PPARA* expression inversely correlated with the expression of human APP (hAPP) protein in AD. We previously demonstrated that increased expression of hAPP in cortical cells inhibits both cholesterol turnover and FA biosynthesis needed for neuronal network activity (23). In addition, we observed that PPARα deficiency leads to decreased LTP (17). Therefore, we have investigated whether modification in *PPARA* expression could be mediated by APP expression levels. We report that *PPARA* mRNA and expression of related PPARα downstream target genes were decreased in transgenic mice and in cultured cortical cells overexpressing nonmutated hAPP, while opposite results were observed in APP-silenced cortical networks. Moreover, APP-mediated effects on *PPARA* expression and thereby on synaptic activity were reversed with PPARα-specific modulators in cultured cortical cells.

## Results

### Human APP–dependent expression of PPARα in brains from patients with LOAD and in early-onset cases with a duplication of the APP locus.

**Figure 1:**
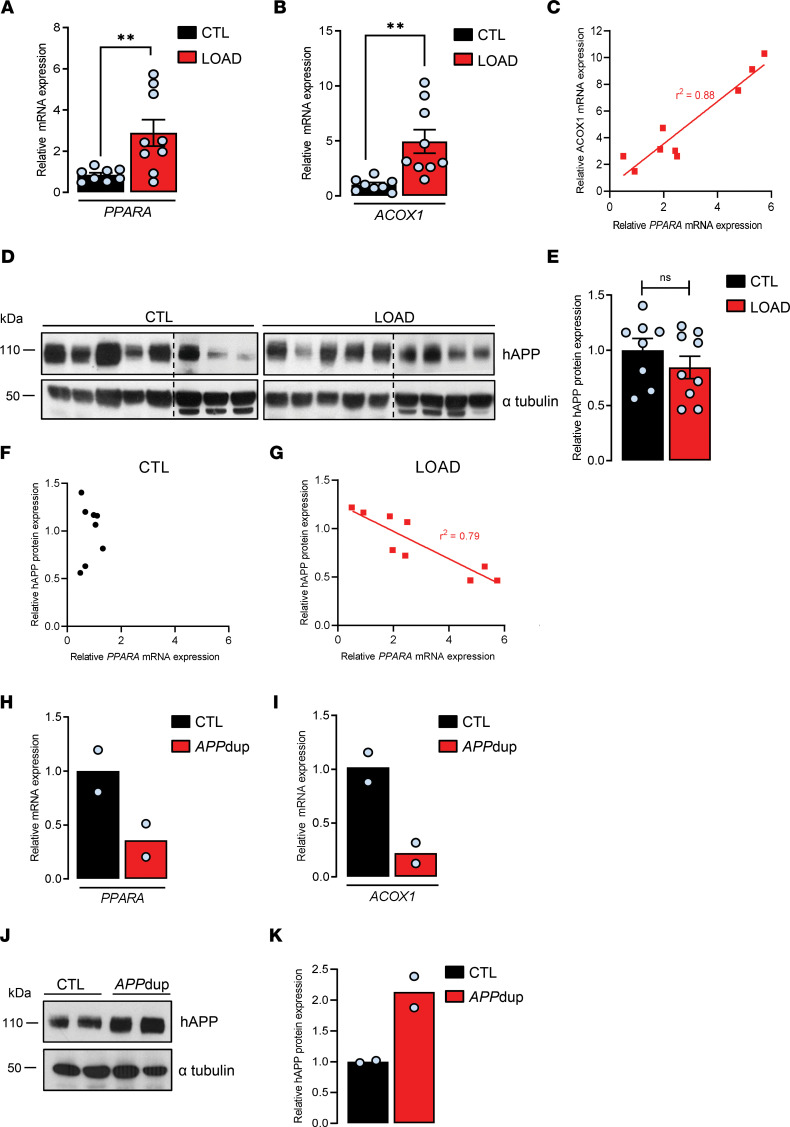
*PPARA* expression and PPARα downstream target genes in brains from patients with AD. Frontal cortex of postmortem human brain tissues from late-onset (LOAD, *n* = 9) and early-onset AD cases with an *APP* duplication locus (*APP*dup, *n* = 2) and respective control subjects (CTL in LOAD and *APP*dup cases, *n* = 8 and 2, respectively) was analyzed. (**A**, **B**, **H**, and **I**) Quantitative real-time PCR analyses for *PPARA* and *ACOX1* mRNA levels. Results were normalized to *ACTB* mRNA, and relative differences are expressed according to respective CTL as mean ± SEM (LOAD: *PPARA* mRNA, *P* = 0.009, *ACOX1* mRNA, *P* = 0.003; Student’s *t* test). (**C**) mRNA correlation between *ACOX1* and *PPARA* in LOAD. (**D** and **J**) Human APP (hAPP) expression in human brain lysates by immunoblot analysis (see complete unedited blots in the supplemental material). (**E** and **K**) Relative density of hAPP expression compared with α-tubulin. Results were normalized compared with respective CTL and are shown as mean ± SEM. A Student’s *t* test (LOAD: hAPP, *P* = 0.0608) was used to assess significance of the mean. (**F** and **G**) Quantification of hAPP densitometry arbitrary units indicating an inverse correlation between hAPP expression and *PPARA* levels in LOAD. **P < 0.01 and nonsignificant (ns) *P* > 0.05.

We first analyzed the expression of *PPARA* mRNA level in human postmortem brains from controls and LOAD cases (Supplemental Table 1; supplemental material available online with this article; https://doi.org/10.1172/jci.insight.150099DS1). A 3-fold increase in the relative expression of *PPARA* (2.88 ± 0.63) was observed in the frontal cortex of LOAD compared with nondemented control samples (Figure 1A). We next analyzed the activation of PPARα in control and LOAD samples by measuring mRNA level of *ACOX1*, the first identified target gene of PPARα that encodes acyl-CoA oxidase 1, a rate-limiting enzyme involved in peroxisomal FA oxidation (11). A 5-fold increase in the relative expression of the *ACOX1* (4.95 ± 1.07) gene was measured in the frontal cortex of LOAD samples compared with controls (Figure 1B). The huge variability in the increased expressions of *PPARA* and *ACOX1* measured in AD brains prompted us to analyze a possible correlation between *PPARA* and *ACOX1* mRNA expressions in LOAD. We observed a strong correlation between PPARα expression (*PPARA* mRNA) and activation (*ACOX1* expression) in LOAD (Figure 1C). Moreover, we observed that the *CPT1A* (4.97 ± 1.37) gene encoding carnitine palmitoyltransferase 1A, a rate-limiting enzyme involved in mitochondrial FA oxidation (11), and *PDK4* (3.83 ± 0.81) encoding pyruvate dehydrogenase kinase 4, which regulates the rate-limiting step of glucose oxidation by switching off the pyruvate dehydrogenase complex (24), were also increased in the frontal cortex of LOAD compared with nondemented control samples (Supplemental Figure 1A). *CPT1A* and *PDK4* are 2 nonspecific PPARα genes known to be nevertheless regulated by PPARα (11). Inasmuch as *ACOX1*, *CPT1A*, and *PDK4* expressions are related to FA and glucose oxidation, these results corroborate abnormalities in brain FA and glucose metabolism, bioenergetics, and mitochondrial function reported in AD (12, 13, 25). Since lipid biochemistry is disrupted in AD (10, 26) and given that one of the main neuropathological characteristics involved in AD pathogenesis implicates the APP (27, 28), which we have previously reported decreases both cholesterol and FA biosynthesis when its expression level increases (23), we wondered whether modifications in *PPARA* expression observed could be related to hAPP expression. While the global expression level of full-length hAPP was unchanged in LOAD compared with controls (Figure 1, D and E), a case-by-case analysis revealed a tight inverse correlation between hAPP and *PPARA* expressions in LOAD brains, but not in control brains (Figure 1, F and G), suggesting that *PPARA* expression is regulated by hAPP expression level only in LOAD. From these results, we next analyzed *PPARA* and PPARα downstream target genes’ expressions in human brain samples from rare EOAD cases with a microduplication in the *APP* locus (Supplemental Table 2) (27, 29). *PPARA*, *ACOX1*, *CPT1A*, and *PDK4* mRNA levels were 3- to 4-fold decreased (0.35 ± 0.15, 0.22 ± 0.09, 0.37 ± 0.10, 0.22 ± 0.15, respectively) (Figure 1, H and I, and Supplemental Figure 1B) in EOAD cases compared with controls, in whom a 2-fold increase in brain hAPP expression (2.13 ± 0.25) was observed compared with age-matched controls (Figure 1, J and K), supporting therefore that increase in hAPP expression was concomitant with decrease in *PPARA*. Altogether these results suggest that nonmutated hAPP expression is inversely correlated with the expression of *PPARA* and PPARα target genes both in LOAD and EOAD cases but not in control human brains.

### Brain Ppara expression and its downstream target genes are decreased in transgenic mice overexpressing WT hAPP.

**Figure 2:**
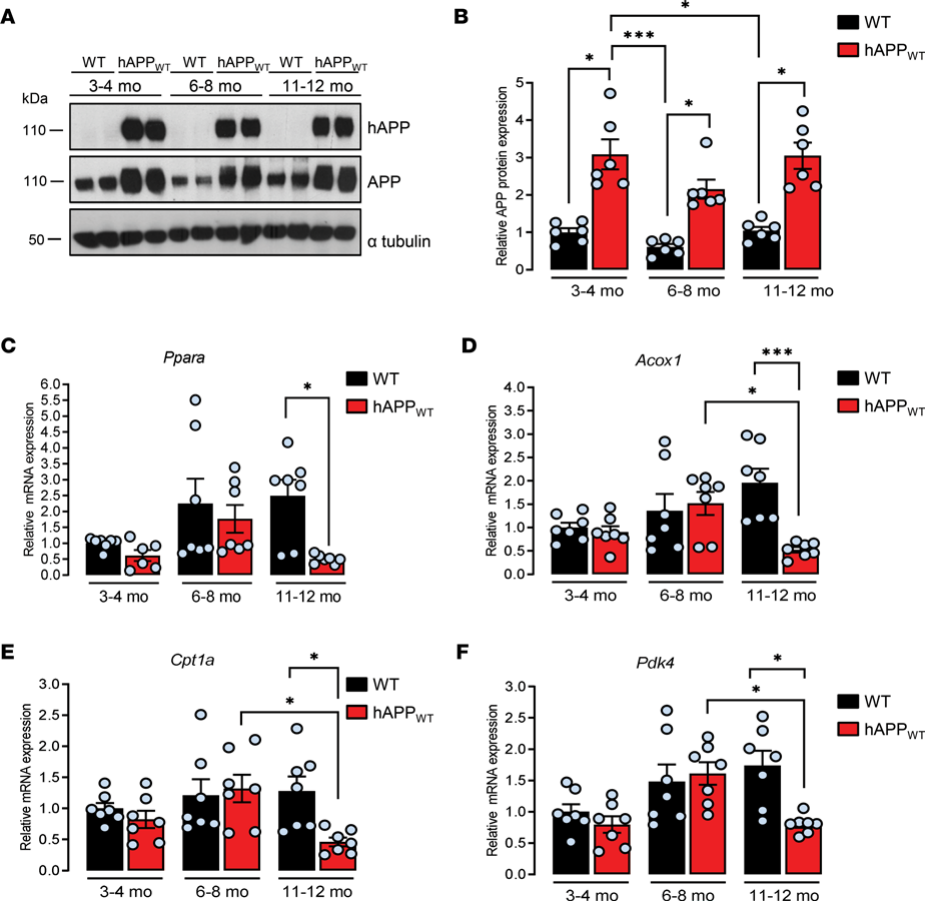
Human APP expression decreases *Ppara* expression and PPARα downstream target genes in old mice. Brain frontal cortex tissues from transgenic mice overexpressing nonmutated human APP (hAPP_WT_) and WT littermates were analyzed at 3–4, 6–8, and 11–12 months old (mo). (**A**) The expression of hAPP was investigated in mice brain lysates (*n* = 6 of each) by immunoblot analysis (see complete unedited blots in the supplemental material) with the specific WO2 antibody recognizing hAPP and anti-APP C-terminal antibody recognizing both hAPP and endogenous APP (APP). Blots were further probed using anti–α-tubulin antibody. (**B**) Relative density of APP expression was compared with α-tubulin. Results were normalized compared with 3–4 mo WT and are shown as mean ± SEM. A Kruskal-Wallis test followed by Dunn’s multiple comparisons posttest was used to assess significance of the mean (APP expression at 3–4, 6–8, and 11–12 mo, *P* < 0.05). (**C**–**F**) Quantitative real-time PCR analyses (*n* = 7 of each) for *Ppara*, *Acox1*, *Cpt1a*, and *Pdk4* mRNA levels. Results were normalized to *Rpl32* mRNA, compared with 3–4 mo WT, and shown as mean ± SEM. A Kruskal-Wallis test followed by Dunn’s multiple comparisons posttest was used to assess significance of the mean (11–12 mo hAPP_WT_ mice: *Ppara* mRNA, *P* = 0.012, *Acox1* mRNA, *P* = 0.0008, *Cpt1a* mRNA, *P* = 0.031; *Pdk4* mRNA, *P* = 0.022), *P < 0.05, ***P < 0.001.

Among transgenic mouse models created to gain insight into AD pathology, the most commonly used are those overexpressing mutated hAPP linked to familial AD (30) resulting in the formation of brain amyloid plaques, one of the pathological hallmarks observed in the brains of patients with AD. Because of their intrinsic relationship, the impact of hAPP expression on *Ppara* expression was separated from that of amyloid deposition by studying the hemizygous transgenic mouse model overexpressing the WT form of hAPP (hAPP_WT_, formerly known as line I5 mouse strain; ref. 31), a mouse model displaying spatial learning and memory deficits with high cortical level of hAPP and no evidence for Aβ deposition (31, 32). By Western blot analysis with an antibody that recognizes human and mouse APP, 2- to 3-fold higher APP levels were observed in hAPP_WT_ as compared with WT mice at early (3–4 months old: 2.67 ± 0.34), advanced (6–8 months old: 1.86 ± 0.21), and late ages (11–12 months old: 2.63 ± 0.30) (Figure 2, A and B) as expected (32). A trend of a higher expression of brain *Ppara* was observed together with a trend of increased *Acox1*, *Cpt1a*, and *Pdk4* mRNA levels along with age in WT mice (Figure 2, C–F), indicating that the aged brain retains enhanced FA utilization compared with young WT mice, meaning that aging brain could switch to FA oxidation (33, 34). While a similar profile was observed in hAPP_WT_ mice at early and advanced ages, a 2- to 5-fold decrease in *Ppara*, *Acox1*, *Cpt1a*, and *Pdk4* mRNA levels was observed (0.46 ± 0.04, 0.58 ± 0.06, 0.50 ± 0.10, 0.91 ± 0.06, respectively) in 11- to 12-month-old hAPP_WT_ mice compared with WT littermates at the same age (Figure 2, C–F), an age at which severe cognitive deficits are detected (32, 35). Metabolic alterations, including deficits in acylcarnitines, have previously been reported in brains of a transgenic mouse model of AD bearing mutated APP and PS1 transgenes, in which early brain amyloid deposition and cognitive impairment occur (36). Nevertheless, our results indicate that in the absence of amyloid deposition, nonmutated hAPP overexpression is sufficient to induce a decrease in brain *Ppara* expression and its downstream target genes and suggest therefore that activation of β-oxidation pathways of FA in the mitochondria might be perturbed in the brains of hAPP_WT_ mice. Moreover, besides hAPP overexpression, an additional age-dependent contribution is needed to observe a decrease in the expression of PPARα downstream target genes, a decrease similar to that previously observed in human brain samples with *APP* duplication.

### Wy14643 PPARα agonist prevents hAPP-induced decreases in PPARα activation and synaptic activity in cortical cultures.

**Figure 3:**
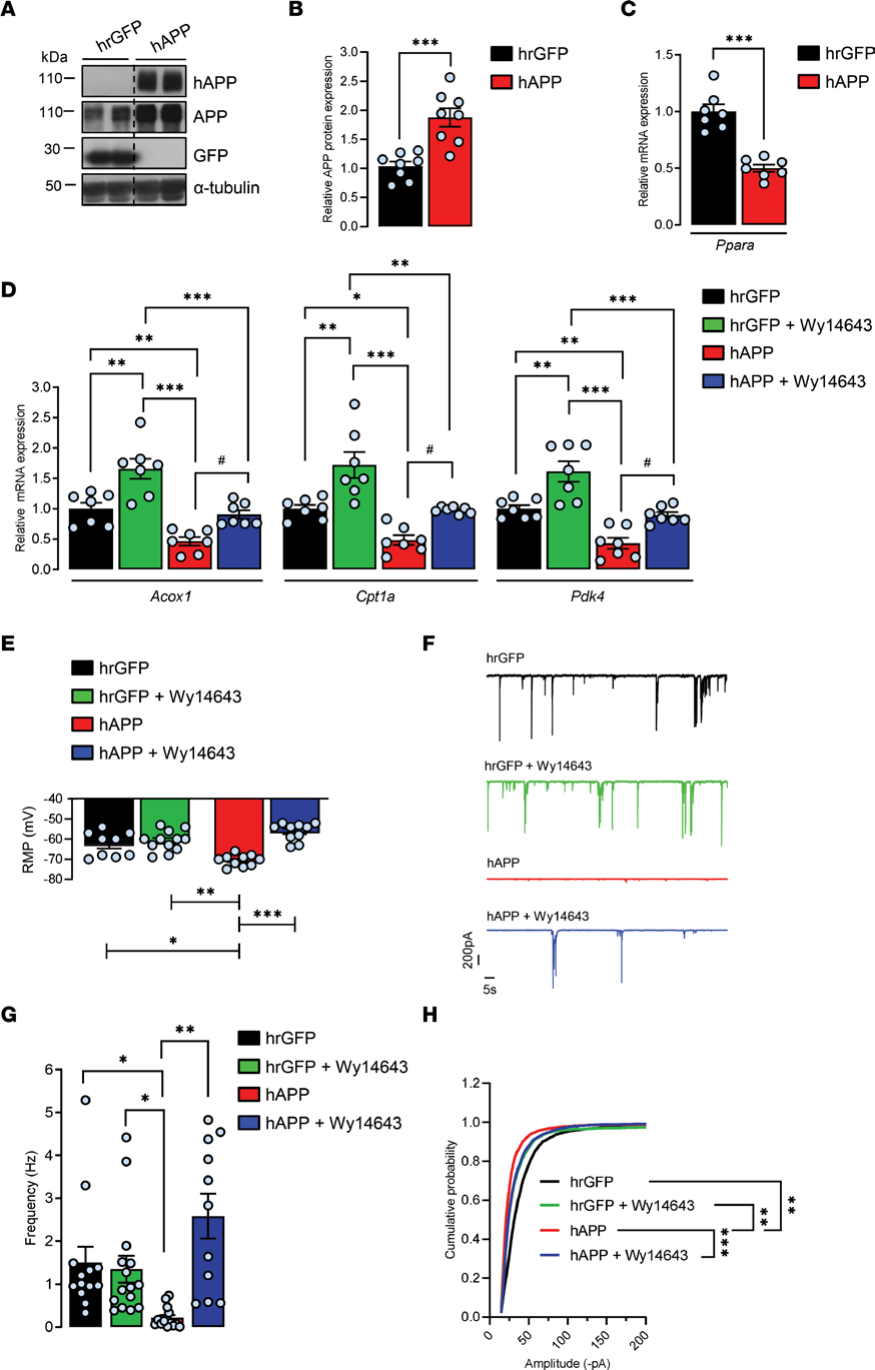
Pharmacological PPARα activation with Wy14643 prevents hAPP-induced decreases in the expression of PPARα target genes and synaptic activity in cortical cultures. Primary cultures of rat cortical cells expressing human recombinant GFP (hrGFP) or hAPP treated (+) or not (-) with 10 μM PPARα agonist Wy14643 for 24 hours at 13–14 DIV. (**A**) Representative immunoblot of cell lysates (4 independent experiments). The lanes were run on the same gel but were noncontiguous. (**B**) APP expression/α-tubulin ratios (*n* = 8 of each analyzed in 4 independent experiments) compared with hrGFP control cells (mean ± SEM); Student’s *t* test (APP, *P* = 0.0608). (**C** and **D**) Real-time PCR analyses for *Ppara*, *Acox1*, *Cpt1a*, and *Pdk4* mRNA levels (*n* = 7 of each analyzed in 4 independent experiments). Results were normalized to *Rpl32* mRNA and compared with respective untreated (-) hrGFP control cells. Results are shown as mean ± SEM; 1-way ANOVA followed by Tukey’s multiple comparisons test: (-) hAPP vs. (-) hrGFP: *Ppara* mRNA, *P* < 0.0001, *Acox1* mRNA, *P* = 0.009, *Cpt1a* mRNA, *P* = 0.026; *Pdk4* mRNA, *P* = 0.003; (+) hAPP vs. (-) hAPP: *Acox1* mRNA, *P* = 0.039, *Cpt1a* mRNA, *P* = 0.035; *Pdk4* mRNA, *P* = 0.022. (**E**) Resting membrane potential (RMP) (*n* = 13–15 cells per group analyzed in 5 independent experiments). (**F**) Representative traces of total synaptic activity and (**G**) mean values of synaptic events’ frequency (*n* = 15–24 cells per group analyzed in 6 independent experiments). (**H**) Cumulative probability plot of the amplitude distribution (*n* = 15–25 cells per group in 6 independent experiments). (**E**–**H**) Brown-Forsythe and Welch 1-way ANOVA tests followed by Dunnett’s T3 multiple comparisons test. *P < 0.05, **P < 0.01, ***P < 0.001, ^#^*P* < 0.05.

Given the finding that activation of all PPARs, but especially PPARγ, has salutary effects on neurodegenerative disorders including AD (19) and that a promising role of PPARα in AD therapy has emerged (10, 37), we wondered whether a PPARα-specific agonist could interfere with the observed hAPP-mediated decrease in PPARα target genes’ expression. To address this question, we expressed neuronal WT hAPP isoform in cultured rat cortical cells. Full-length hAPP expression was achieved by transducing primary cortical cultures with a recombinant adenoviral vector at 6 days in vitro (DIV), and hAPP expression was probed by immunoblotting at 13–14 DIV with the hAPP-specific WO2 antibody (Figure 3A). We have previously shown that transgene expression is homogenous, does not affect cell density and viability, and remains stable over time (38). By using an antibody that recognizes both human and endogenous rat APP, a nearly 2-fold increase in total APP levels (1.87 ± 0.15) was observed compared with control cells transduced with an adenovirus encoding the hrGFP (Figure 3, A and B). As previously observed in human and mouse brain lysates overexpressing hAPP, *Ppara* (0.49 ± 0.05), *Acox1* (0.40 ± 0.06), *Cpt1a* (0.39 ± 0.06), and *Pdk4* (0.46 ± 0.10) mRNA levels were reduced by about 60% in hAPP-expressing cells compared with hrGFP vehicle–treated controls (Figure 3, C and D). It is very well-known that cancers reprogram their metabolism to adapt to environmental changes. Cpt1a fuels lipid β-oxidation by producing acyl-carnitines in the mitochondria (39). It was recently demonstrated that in prostate cancer cell models, overexpression of *Cpt1a* is associated with a significant increase in intracellular lipase activity (40), a step that liberates fatty acids from triglyceride stores, which can then be used for β-oxidation (41). Consequently, overexpression of *Cpt1a* increases free fatty acid (FFA) content in these cancer cells. We measured whether modification in Cpt1a expression influences FFA content in our cultured cortical cells. In hAPP-expressing cells, a decrease in *Cpt1a* expression was concomitant with a 70% decrease in cell FFA content (0.32 ± 0.06) compared with hrGFP control (Supplemental Figure 2A), demonstrating that modification in *Cpt1a* expression affects FA metabolism in our cortical cells in culture. Moreover, since Pdk4 is known to play a role in the metabolic switch from glycolysis to mitochondrial oxidative phosphorylation (OXPHOS) (24), the decrease in *Pdk4* observed indicates that hAPP expression enhances glycolysis in cortical cells (42). Cells were then treated with pirinixic acid (Wy14643), a synthetic PPARα agonist (reviewed in refs. 19, 43). As expected, the basal expression of *Acox1* (1.66 ± 0.16), *Cpt1a* (1.72 ± 0.21), and *Pdk4* (1.61 ± 0.16) genes was increased in Wy14643-treated hrGFP cells compared with vehicle-treated cells (Figure 3D). Moreover, Wy14643 was able to inhibit the hAPP-mediated decrease in *Acox1*, *Cpt1a*, and *Pdk4* mRNA levels (Figure 3D), indicating that the pharmacological modulation of PPARα is able to restore hAPP-mediated effects on PPARα target genes’ expression in cortical cells in culture.

As PPARα deficiency affects neuronal activity in cultured hippocampal neurons (16) and synaptic plasticity in mice (17), we hypothesized that decrease in PPARα target genes’ expression could contribute to the hAPP-mediated synaptic transmission silencing of cortical networks (23, 44, 45). To study the effect of 5–7 days’ hAPP expression on synaptic activity, we performed whole-cell voltage clamp recordings. Resting membrane potential (RMP) was more negative (ΔVm –8.04 mV ± –1.16) (38) (Figure 3E), and concomitant decreases in spontaneous total synaptic activity (Figure 3F), frequency (hrGFP, 1.50 ± 0.37 Hz; hAPP, 0.21 ± 0.06 Hz; Figure 3G), and amplitude averages (hrGFP, 32.41 ± 1.30 pA; hAPP, 20.93 ± 1.22 pA, Figure 3H) were measured in hAPP neurons compared with hrGFP controls. However, Wy14643 treatment partially prevented the effect of hAPP expression on synaptic activity (Figure 3F), restored RMP, and increased both frequency (vehicle hAPP, 0.21 ± 0.06 Hz; Wy14643 hAPP, 2.58 ± 0.51 Hz) and amplitude of synaptic events (vehicle hAPP, 20.93 ± 1.22 pA; Wy14643 hAPP, 27.79 ± 0.67 pA), while no significant changes were observed in Wy14643 hrGFP compared with vehicle-treated controls (Figure 3, E, G, and H). Altogether, these results indicate that PPARα activation with a specific agonist can prevent hAPP-mediated synaptic activity depression of cortical networks.

### GW6471 PPARα antagonist inhibits APP knockdown–induced increases in PPAR activation and synaptic activity in cortical cultures.

**Figure 4:**
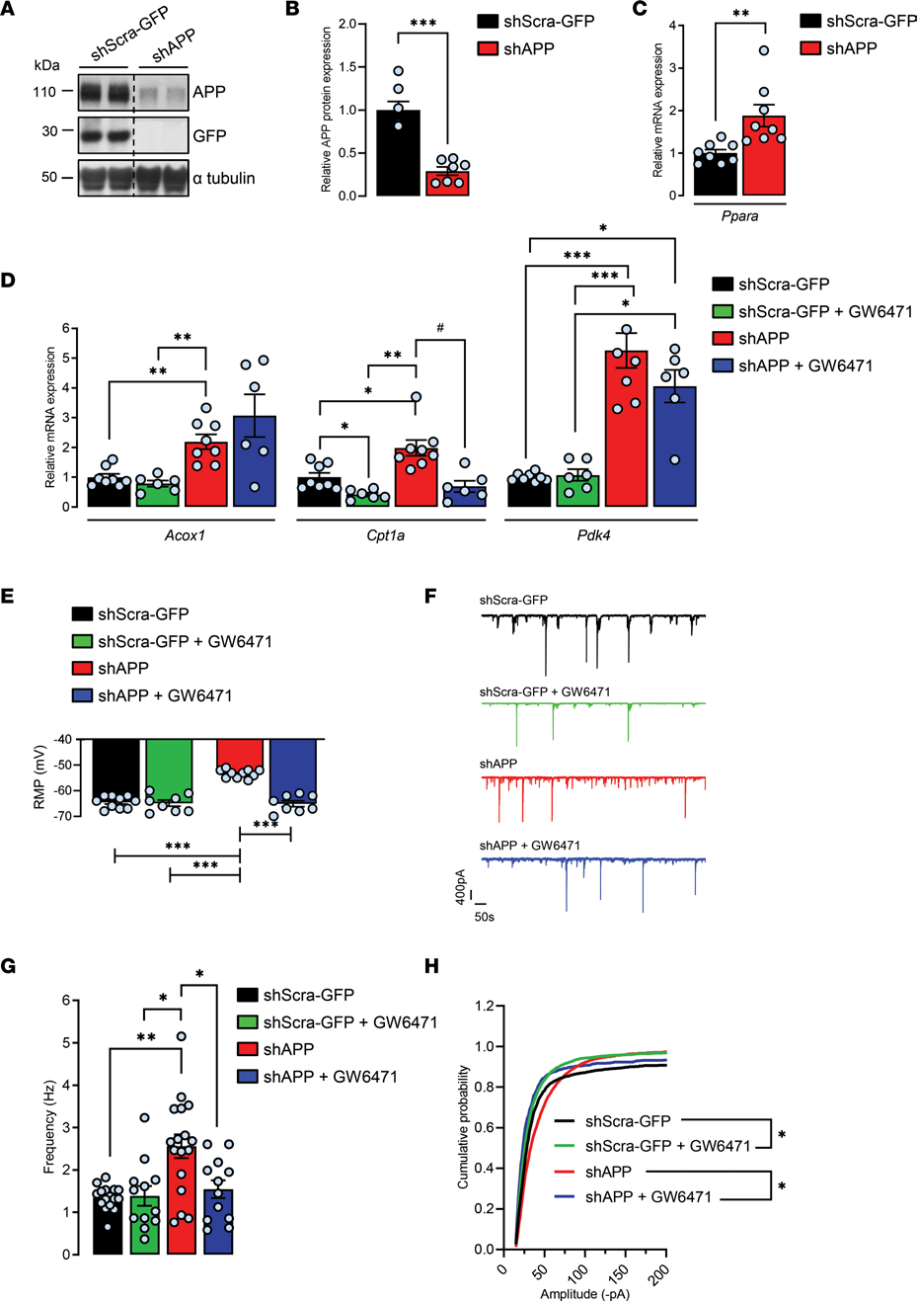
Pharmacological PPARα inhibition with GW6471 prevents APP knockdown–induced increases in the expression of PPAR target genes and synaptic activity in cortical cultures. Primary cultures of rat cortical cells expressing a shRNA targeting endogenous APP (shAPP) or a scrambled shRNA encoding GFP (shScra-GFP). At 13–14 DIV, cells were treated (+) or not (-) with PPARα antagonist GW6471 for 24 hours. (**A**) Representative immunoblot of cell lysates, 4 independent experiments (the lanes were run on the same gel but were noncontiguous). (**B**) APP expression/α-tubulin ratios (*n* = 7 of each analyzed in 4 independent experiments), mean ± SEM; Student’s *t* test (APP, *P* < 0.001). (**C** and **D**) Real time PCR analyses for *Ppara*, *Acox1*, *Cpt1a*, and *Pdk4* mRNA levels (*n* = 6–8 for each condition analyzed in 6 independent experiments). Results were normalized to *Rpl32* mRNA and compared with respective untreated (-) shScra-GFP control cells. Results are shown as mean ± SEM; Brown-Forsythe and Welch 1-way ANOVA tests followed by Dunnett’s T3 multiple comparisons test: (-) shAPP vs. (-) shScra-GFP: *Ppara* mRNA, *P* = 0.006, *Acox1* mRNA, *P* = 0.008, *Cpt1a* mRNA, *P* = 0.043; *Pdk4* mRNA, *P* = 0.0009; (+) shScra-GFP vs. (-) shScra-GFP: *Cpt1a* mRNA, *P* = 0.026; (+) shAPP vs. (-) shAPP: *Cpt1a* mRNA, *P* = 0.010. (**E**) RMP (*n* = 8–10 cells per group analyzed in 3 independent experiments). (**F**) Representative traces of total synaptic activity and (**G**) mean values of synaptic events’ frequency (*n* = 12–17 cells per group analyzed in 6 independent experiments). (**H**) Cumulative probability plot of the amplitude distribution (*n* = 15–27 cells per group in 7 independent experiments). (**E**–**H**) Brown-Forsythe and Welch 1-way ANOVA tests followed by Dunnett’s T3 multiple comparisons test. *P < 0.05, **P < 0.01, ***P < 0.001, ^#^*P* < 0.05.

To further investigate the possible modulation of PPARα activation by APP, APP expression was reduced in cortical cells by using shRNA construct designed to target endogenous APP (shAPP). The knockdown of endogenous rat APP was achieved by transducing cells at 6 DIV with recombinant lentiviruses encoding shAPP, and a scrambled shRNA encoding GFP (shScra-GFP) was used as a negative control. At 13–14 DIV, blots were probed with an antibody that recognizes the 19 carboxyl terminal amino acids of endogenous APP (Figure 4A). As expected, a 66.66% ± 5.7% reduction of endogenous APP expression (38) (Figure 4B) with no induction in the expression of APLP1 and APLP2 for functional compensation for the loss of APP was observed as we have previously reported (23, 38). Unlike hAPP cells, APP knockdown induced a 2-fold increase in *Ppara* (1.89 ± 0.26), *Acox1* (2.19 ± 0.24), and *Cpt1a* (1.98 ± 0.18) and robustly increased *Pdk4* (4.22 ± 0.63) mRNA levels compared with controls (Figure 4, C and D), indicating that PPARα activation was increased in these cells. Contrary to hAPP-expressing cells, an increase in *Cpt1a* expression was concomitant with a 75% increase in FFA content (1.75 ± 0.10) in shAPP cells compared with shScra-GFP control cells (Supplemental Figure 2B). These results confirm that APP-mediated modification in *Cpt1a* expression affects FA metabolism in cortical cells. Moreover, these results suggest that reduction in endogenous APP enhances the transport of FAs into the mitochondria for β-oxidation and that shAPP cells may be less glycolytic (46). We next used GW6471 to specifically inhibit PPARα activity (47) in order to counteract the shAPP-mediated effect on PPARα activation. The effectiveness of GW6471 was shown by the decreased expression of *Cpt1a* observed in both treated shAPP (0.69 ± 0.18) and shScra-GFP (0.41 ± 0.06) cells compared with vehicle-treated controls (Figure 4C), while no changes were observed in *Acox1* and *Pdk4* mRNA levels. These results indicate that the GW6471 PPARα antagonist partially inhibits APP knockdown–induced increase in PPARα activation and specifically affects *Cpt1a* expression in cortical cells.

To address whether increase in PPARα activation could contribute to shAPP-mediated increase in neuronal activity of cortical networks as reported (23), we performed whole-cell voltage clamp recordings in APP-knockdown neurons and treated them with GW6471. RMP was more positive (ΔVm –11.20 ± –0.22 mV) (Figure 4E), and concomitant increase in total synaptic activity (Figure 4F) frequency (shScra-GFP, 1.30 ± 0.06 Hz; shAPP, 2.55 ± 0.27 Hz; Figure 4G), with no significant change in the amplitude (shScra-GFP, 51.89 ± 10.49 pA; shAPP, 51.42 ± 3.09 pA, Figure 4H), was measured in shAPP compared with shScra-GFP controls. Moreover, GW6471 restored RMP, frequency, and amplitude in treated shAPP to levels similar to vehicle-treated controls (Figure 4, E–H). Altogether, these results indicate that APP expression levels modulate the activation of PPARα and thereby synaptic function and that pharmacological approaches targeting PPARα allow us to reverse APP-mediated effects observed in cortical cells in culture.

### Control of synaptic activity by APP disappears in the absence of PPARα in cortical cultures.

**Figure 5:**
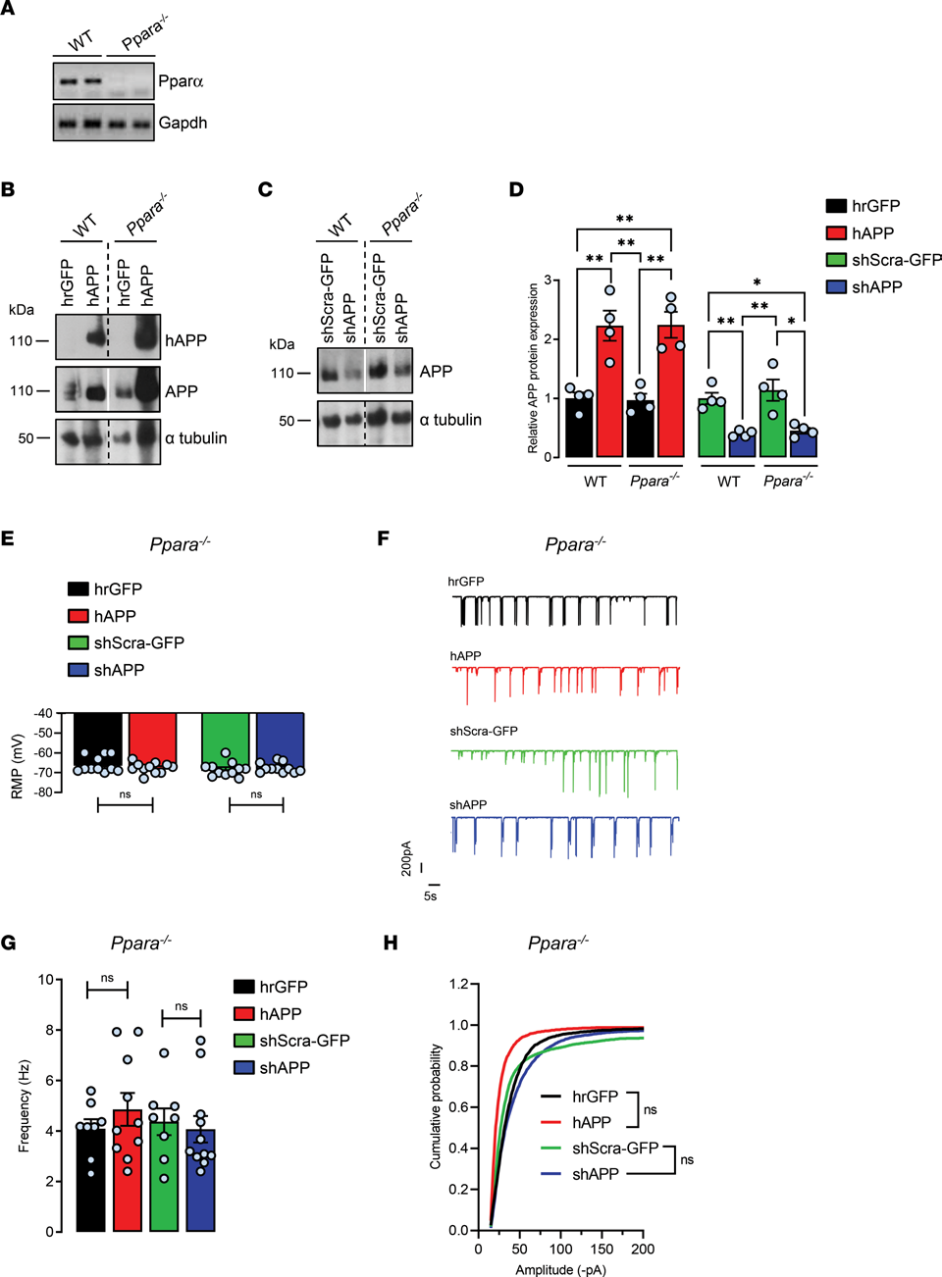
Control of synaptic activity by APP in cortical cultures disappears in the absence of PPARα. Primary cultures of mouse cortical cells prepared from WT and *Ppara*-deficient (*Ppara^–/–^*) mice and infected with recombinant adenoviruses encoding hrGFP or hAPP proteins or with lentiviruses encoding a shRNA construct designed to target endogenous APP (shAPP) or a scrambled shRNA encoding GFP (shScra-GFP). (**A**) At 13–14 DIV, absence of PPARα expression in cultured cells was assessed by measuring *Ppara* mRNA levels by semiquantitative real-time PCR (RT-PCR). (**B** and **C**) Representative immunoblots of cell lysates, 3 independent experiments (the lanes were run on the same gel but were noncontiguous). The expression of hAPP was monitored with the specific WO2 antibody recognizing hAPP and anti-APP C-terminal antibody recognizing both hAPP and endogenous APP (APP). Immunoblots were further probed using anti-GFP and –α-tubulin antibodies. (**D**) APP expression/α-tubulin ratios (*n* = 4 of each) compared with hrGFP or shScra-GFP WT cells (mean ± SEM); 1-way ANOVA followed by Tukey’s multiple comparisons test (APP: WT and *Ppara^–/–^* hAPP *P* = 0.002; WT and *Ppara^–/–^* *s*hAPP, *P* = 0.008 and *P* = 0.015, respectively). (**E**) RMP measured in WT and *Ppara^–/–^* transduced neurons (*n* = 11 cells per group analyzed in 3 independent experiments). (**F**) Representative traces of total synaptic activity and (**G**) mean values of synaptic events’ frequency (*n* = 8–11 cells per group analyzed in 3 independent experiments). (**H**) Cumulative probability plot of the amplitude distribution (*n* = 10–20 cells per group in 3 independent experiments) measured in WT and *Ppara^–/–^* transduced neurons. (**E**–**H**) Brown-Forsythe and Welch 1-way ANOVA tests followed by Dunnett’s T3 multiple comparisons test. *P < 0.05, **P < 0.01, and ns *P* > 0.05.

To address whether APP-mediated control of synaptic activity could be PPARα dependent, primary cultures of cortical cells derived from WT and PPARα-deficient (*Ppara^–/–^*) mice (Figure 5A) were transduced with recombinant viruses in order to express hAPP (Figure 5B) or to repress endogenous APP (Figure 5C). With a similar extent as observed previously in rat primary cultures, a 2-fold increase (2.22 ± 0.25) and a 60% reduction (0.40 ± 0.02) in total APP levels were observed in hAPP and shAPP WT relative to infected WT controls. Moreover, PPARα deficiency did not affect the total APP content increase (2.24 ± 0.22) and decrease (0.45 ± 0.05) observed in hAPP and shAPP *Ppara^–/–^* compared to hAPP and shAPP WT, respectively (Figure 5D). Although the knockout of PPARα increased averages of total synaptic activity frequency (4.34 ± 0.51 Hz) and amplitude (64.47 ± 3.45 pA) in all infected conditions, PPARα deficiency totally prevented APP-mediated effects on RMPs, synaptic event frequencies, and amplitudes recorded in hAPP and shAPP *Ppara^–/–^* compared, respectively, with hrGFP and shScra-GFP controls (Figure 5, E–H). Altogether, these results suggest that PPARα is a downstream mediator of APP-mediated control on synaptic activity.

## Discussion

We report here that *PPARA* expression and PPARα downstream target genes were inversely correlated with hAPP expression in both LOAD and EOAD with a microduplication of the *APP* locus. Such an effect of hAPP expression was also observed in hAPP-transgenic mice and in cortical networks, in which pharmacological approaches targeting PPARα alleviated APP-mediated synaptic dysfunction.

The overall increase in the expression of *PPARA* mRNA that we have observed in LOAD is not in agreement with previous results reporting globally reduced expression level of PPARα in AD brains (22). However, a case-by-case analysis reveals a tight inverse correlation between hAPP and *PPARA* expressions in our LOAD samples, but not in control brains, suggesting that *PPARA* expression is regulated by hAPP expression level only in LOAD. The large variability of both hAPP and *PPARA* mRNA contents observed in the LOAD group could probably account in part for the discrepancy with the results published by de la Monte and colleagues (22). Expression, trafficking, and processing of APP are regulated in a complex way, including prominent changes during pathological states. It was previously reported that APP expression is upregulated under conditions of metabolic stress (48), ischemia (49), brain injury (50), and inflammation (51) and that individual APP expression is heterogeneous in patients with AD (49). Moreover, our results put forward that APP expression seems to play an important role on *PPARA* expression in LOAD, but not in controls, in which no correlation between hAPP and *PPARA* mRNA contents was observed. Furthermore, our results demonstrate that in addition to *PPARA* gene expression, expression of *ACOX1*, *CPT1A*, and *PDK4* PPARα target genes is inversely correlated with hAPP expression when LOAD samples are analyzed individually. This inverse correlation between hAPP and *PPARA* expression and activation is confirmed in EOAD cases with a rare duplication of the *APP* locus. We conclude that particular attention should be paid to the level of hAPP expression in each AD case studied when the expression of *PPARA* is considered. Such an inverse correlation between hAPP and *PPARA* expression was not observed in control brains, indicating a specific function of hAPP in AD.

In the brains of AD patients, microglial activation is observed in the vicinity of senile plaques at all stages of the disease and is accompanied by increased levels of proinflammatory molecules (e.g., TNF, IL-1β, IL-6, prostaglandins) (52). Genome-wide association studies identified inflammation-related genes as potential risk factors for developing AD (53). In addition, it was recently reported that APP expression is increased under inflammatory processes in an AD transgenic mouse model (51). Therefore, we cannot exclude that inflammation could mediate changes in brain APP expression that could thereby affect *PPARA* expression in AD samples analyzed.

Increases in *ACOX1* and *CPT1A* observed in LOAD with a low hAPP content indicate that β-oxidation pathways of very long-chain FAs in the peroxisome and short-, medium-, and long-chain FAs in the mitochondria are activated. Activation of FA oxidation might take place to compensate for compromised pyruvate dehydrogenase complex (PDC) to provide alternative sources of acetyl-CoA to sustain ATP energy supply. Indeed, a concomitant increase in the expression of *PDK4* that catalyzes the phosphorylation-dependent inactivation of the mitochondrial PDC (24) was observed in LOAD, in whom a reduction in PDC activity was previously reported (54). As mitochondrial PDC connects glycolysis to oxidative metabolism (55) and as PDK4 upregulation facilitates FA oxidation, in particular when glucose is scarce during energy deprivation (56), the *PDK4* increase observed in LOAD brains correlates with the reduced cerebral glucose utilization found in patients with AD (12), in which early brain peroxisomal and mitochondrial function deficits have been reported (reviewed in refs. 22, 57). Although a shift in brain metabolism from glucose-driven energy supply to a ketogenic/FA oxidation pathway is reported in LOAD (58), this shift could depend on the level of APP expression. In addition, an opposite shift may take place in EOAD carrying a microduplication of the *APP* locus (27, 29). Indeed, an inverse correlation was observed in brain samples from *APP*dup cases, in which increase in hAPP is concomitant with decreases in *PPARA*, *ACOX1*, *CPT1A*, and *PDK4* expression. We conclude that metabolic shifts observed in LOAD and EOAD could rely on hAPP expression level, suggesting that hAPP by controlling *PPARA* expression and its downstream target genes might be considered an essential metabolic mediator in AD but not in control brains. Although modifications in brain *PPARA*, *ACOX1*, *CPT1A*, and *PDK4* expression observed could be mediated by variations in hAPP expression in AD, a role for hAPP cleavage products including Aβ, tau phosphorylation, brain inflammation, synaptic loss, and amyloid burden reported in AD brains cannot be ruled out (2).

Interestingly, neuropathological changes observed in AD were reported in patients with Down syndrome (DS), in whom the presence of an extra chromosome 21 leads to intellectual disability. Association between AD and DS is partially due to the overexpression of hAPP that results from the location of *APP* gene on chromosome 21 (59). Moreover, mitochondrial deficits, increase in oxidative stress, and impaired glucose and lipid metabolism leading to a reduced rate of energy metabolism were reported in DS (reviewed in ref. 60). Recently, severe brain malformations with pyruvate dehydrogenase deficiency and DS were reported (61), and the Down syndrome critical region 2 protein was shown to inhibit the transcriptional activity of PPARβ in a cell line, indicating a potential dysfunction of PPAR activation in DS, in which hAPP expression level is increased (62).

APP-dependent modulation of brain *PPARA*, *ACOX1*, *CPT1A*, and *PDK4* expression was observed not only in human AD brain but also in mice. Indeed, we show that overall increase in APP expression lowers brain *Ppara* expression and thereby *Acox1*, *Cpt1*, and *Pdk4* in hAPP_WT_ mice at late stages (31, 32). An increase in Acox1 expression was found in the hippocampus of young Tg2576 mice, a mouse model of AD harboring the human Swedish familial AD mutation that develops parenchymal amyloid plaques at 11–13 months of age, while Acox1 expression was found to be decreased in old animals (63, 64). Moreover, *Pdk4* decrease observed in old hAPP_WT_ mice corroborates the decrease in the PDC reported in brain synaptosomes of Tg2576 mice (65). However, decreases in *Acox1* and *Pdk4* observed in APP_WT_ mice rule out a possible involvement of amyloid plaques in these mice, which are devoid of brain Aβ deposition (31, 32).

APP-dependent regulation of *Ppara*, *Acox1*, *Cpt1a*, and *Pdk4* mRNA observed in hAPP_WT_ mice, in EOAD with a duplication of the *APP* locus, and in LOAD cases was recapitulated in hAPP-expressing and APP-silenced cultured cortical cells, indicating a critical involvement of APP expression in the regulation of *Ppara* and its downstream target genes. The diminution of *Pdk4* observed in hAPP-expressing cells indicates that hAPP expression and/or increase in total APP content enhances glycolytic metabolism, as reported in human neuroblastoma cells in which increase in neuronal hAPP levels mediates an Aβ-independent Pdk4 downregulation (42). Conversely, the robust *Pdk4* increase observed in APP-silenced cortical cells suggests that APP reduction lowers cell glycolytic capacity (46). Variations in APP expression therefore modulate *Ppara* expression and its downstream target genes in cultured cortical cells, strengthening a potential role of APP expression as metabolic mediator. Moreover, a pivotal role of Pdks and metabolic flexibility was reported in the brain, which utilizes glucose as a primary energy source (24). Astrocytes express more Pdks than neurons and have lower PDC activity (66), indicating that APP-mediated changes in Pdk4 expression might occur primarily in astrocytes in our cultured cells. Moreover, the increase in *Cpt1a* observed in APP-knockdown cells takes place probably in astrocytes also (33), in which FA oxidation predominantly occurs to contribute up to 20% of the total brain energy requirement as reported in brain primary cultures (67). Given the tight metabolic coupling between astrocytes and neurons, a metabolic transition from glycolysis to OXPHOS could occur in APP-knockdown cells to provide adequate ATP level to meet the increased energy demand needed for the sustained synaptic activity observed.

While we observed a depressive effect of hAPP expression on synaptic transmission that could result from an Aβ-dependent postsynaptic silencing of α-amino-3-hydroxy-5-methyl-4-isoxazolepropionic acid (AMPA) receptor– (45) and *N*-methyl-d-aspartate (NMDA) receptor–mediated currents (44), that synaptic excitatory transmission increased in APP-silenced neurons suggests that changes in neuronal activity drive changes in metabolic flux or vice versa. While energy metabolism of neurons is mainly aerobic and that of astrocytes mainly anaerobic glycolysis, OXPHOS was shown to be the main mechanism initially providing energy to power neuronal activity (68). Accordingly, miniature excitatory postsynaptic currents’ frequency was shown to be increased in neurons derived from APP-knockout mice (69), in which a resistance to an HFD-induced obesity was observed and linked to higher energy expenditure and lipid oxidation (70). Moreover, changes in synaptic activity observed could be mediated by the Cpt1c isoform. Indeed, despite the inability of the brain-specific Cpt1c isoform to β-oxidize long-chain FAs contrary to Cpt1a (71), Cpt1c could enhance whole-cell currents of shAPP neurons by increasing the trafficking and the surface expression of the GluA1 subunit containing AMPA receptors to enhance AMPA receptor–mediated currents (71).

Our findings also put forward that APP-mediated changes in the expression of *Acox1*, *Cpt1a*, and *Pdk4* are driven by metabolic regulators and in particular PPARα. The central role of PPARα in FA catabolism is very well-known (11). PPARα increases expression of genes encoding peroxisomal and mitochondrial enzymes, including Acox1 and Cpt1, and both PPARα and PPARβ/δ but not PPARγ also activate the Pdk4-encoding gene (11). We report that PPARα modulators were able to reverse APP-mediated effects observed in cultured cortical cells. PPARα synthetic agonist Wy14643 normalized the expression of PPARα target genes and restored synaptic activity depressed in hAPP-expressing cells. This could result from the proliferation of peroxisomes and/or the expression of peroxisomal enzymes that prevent Aβ-mediated cell death and/or oxidative stress as reported in rat hippocampal and cortical cultures, respectively (72, 73). Moreover, PPARα agonists have been reported to promote the nonamyloidogenic processing of APP in hippocampal neurons by enhancing the expression of the α-secretase ADAM10 that precludes Aβ generation from APP and increases the release of the soluble APP α-fragment (74). Therefore, PPARα agonist Wy14643 could promote the nonamyloidogenic processing of APP, alleviating therefore the Aβ-mediated negative feedback on synaptic transmission observed in hAPP neurons.

PPARα plays a key role in inflammation, and PPAR agonists are antiinflammatory drugs targeting microglia and astrocytes (11). However, activation of PPARα also produces a strong neuronal signature, by regulating glutamatergic and cholinergic mediated dopaminergic transmission in the brain (75). Although our cortical cells in culture contained both neurons and astrocytes, patch clamp analyses were performed exclusively on neurons, confirming that modulation of the activity of PPARα influenced neuronal activity.

Furthermore, we report that PPARα antagonist GW6471 inhibited APP knockdown–induced increases in PPAR activation and synaptic activity in cortical cultures. GW6471 decreased the expression of upregulated *Cpt1a* without modifying the expression of *Acox1* and *Pdk4* of shAPP cells, pointing therefore to differences between Wy14643 and GW6471 in their binding affinity and/or in the recruitment of PPARα nuclear coactivators and/or corepressors (47). However, we report that GW6471 was able to normalize intensive synaptic activity of shAPP cells. The knockdown of APP in neuronal precursor cells of the hippocampus was previously reported to affect synaptic GluN2B-containing NMDA receptors (76) and PPARα, but not PPARβ, and PPARγ was shown to regulate cyclic AMP response element binding and therefore hippocampal plasticity-related genes encoding GluN2A/2B and GluA1 subunits of NMDA and AMPA receptors (16, 17). From these observations, GW6471 could then affect both GluN2B and GluA1 expressions and/or Cpt1c-mediated trafficking of GluA1 (71) to modulate synaptic activity of shAPP neurons. Furthermore, we report that deficiency of PPARα in cortical cells abrogates APP-mediated controls of synaptic activity, confirming that PPARα is an APP downstream mediator.

A growing body of evidence suggests that synaptic dysfunction may occur long before synapse loss in early AD and may therefore contribute to cognitive dysfunction (3). Abnormalities in brain activity have been reported in both LOAD and EOAD (reviewed in ref. 77), in which an increased incidence of seizures has been observed, and greater risk for seizures was previously recorded in patients with *APP* duplication and in DS with dementia (78, 79). Spontaneous seizures and sharp wave discharges have been observed in several transgenic models of AD expressing APP (harboring or not harboring mutations) (32). Moreover, APP overexpression, but not a subsequent Aβ increase, leads to hypersynchronous network activity in an APP-transgenic mouse model of AD, suggesting that APP overexpression elicits network alterations through an indirect mechanism (80). Although salutary effects of PPARα and -γ agonists on memory have been reported in several preclinical AD models that overexpress APP, human clinical trials using PPARγ agonists in the treatment of AD are less encouraging (reviewed in refs. 10, 37). A U-shaped relationship between APP level and its functions has been put forward given that mice overexpressing or lacking APP exhibit, inter alia, age-dependent deficits in LTP and learning, an activity-dependent enhancement of synaptic strength involved in memory processing (18), vulnerability to seizures, and metabolic stress (reviewed in ref. 77).

As cell energy metabolism and synaptic activity are closely related, it is still questionable whether a PPARα agonist or antagonist could help for synaptic abnormalities associated with cognitive impairments observed in AD. However, our results put forward that the expression of APP as a metabolic regulator should be considered throughout the course of the disease, in which potential APP-mediated metabolic switches driven by PPARα could occur. Moreover, overlapping metabolic dysfunctions reported in AD and metabolic diseases such as obesity and type 2 diabetes (for a review, see ref. 10) that have been identified as AD risk factors emphasize an essential role of lipid and glucose metabolism in the etiology of AD. Therefore, pharmacological modulation of the PPARα metabolic regulator could be part of a personalized multitherapy that could help patients with AD, depending on their level of APP expression.

## Methods

For cell culture, semiquantitative RT-PCR, immunoblotting analysis, recombinant viruses, and cell transduction, an extended section is provided in Supplemental Methods.

### Human tissues and animals

#### Human tissues.

Frontal cortex samples from 10 human control subjects and 11 demented patients were analyzed. All patients were clinically diagnosed. Neuropathological examinations were performed on multiple formalin-fixed, paraffin-embedded postmortem frozen brain tissues and confirmed clinically diagnosed patients as having LOAD cases. Genetic analyses were performed on patients with EOAD with *APP* microduplication mapping to chromosome 21q2.1, including the APP locus with no contiguous gene. An overview of the donor information and postmortem variables is summarized in Supplemental Tables 1 and 2.

#### Animals.

Pregnant Wistar rats were obtained from the Catholic University of Louvain animal facility (Brussels, Belgium), and P0–P1 pups from PPARα-deficient (*Ppara^–/–^*) mice (JAX stock 008154) were utilized (81) for embryonic rat and mouse cortical cell cultures, respectively. Age-matched nontransgenic WT littermates were used as controls. For *Ppara^–/–^* mice, genotypes were confirmed by PCR amplification from mouse tail biopsy DNA using KAPA Express Extract combined with KAPA2G Robust HotStart Ready Mix (KK7152) by using the following standard Jackson Laboratory–suggested primers (see Supplemental Table 3).

We used transgenic male mice, 3–4, 6–8, and 11–12 months old, expressing a transgene containing WT human APP (APP_WT_ line I5 mouse strain, ref. 31, JAX stock 004662). Genotypes were confirmed by PCR amplification with the following standard Jackson Laboratory–suggested primers (see Supplemental Table 3). Mice were group-housed under standardized conditions (12-hour light/12-hour dark cycle, not reversed), with free access to food (SAFE A03, SAFE Diets) and water ad libitum. Temperature in the vivarium was maintained between 20°C and 24°C and humidity between 45% and 65%.

#### Brain mouse tissue collection.

Brains from transgenic mice were snap-frozen in liquid nitrogen and stored at −80°C until further use (RNA isolation for RT-PCR and Western blotting).

### Reagents and antibodies

When unmentioned, reagents for cell culture and Western blotting were purchased from Thermo Fisher Scientific. Antibodies were purchased as indicated in Supplemental Table 4.

### Treatments

At 13–14 DIV, cells were treated for 24 hours with 10 μM Wy14643 (Tocris, Bio-Techne, 1312), 5 μM GW6471 (Tocris, Bio-Techne, 4618) or vehicle (0.0001% and 0.005% DMSO, respectively). For electrophysiology, cells were treated with 1 μM GW6471.

### RNA extraction and RT-PCR

Total RNA was isolated by TriPure Isolation Reagent (Roche, 11667165001) according to the manufacturer’s protocol. RNA samples were resuspended in DEPC-treated water (1 μg/10 μL). Reverse transcription was carried out with the iScript cDNA Synthesis Kit (Bio-Rad Laboratories, 1708891) using 1 μg of total RNA in a total volume reaction of 20 μL. Real-time PCR was performed for the amplification of cDNAs with specific primers (MilliporeSigma, see Supplemental Table 5). RT-PCR was carried out in a total volume of 25 μL containing 16 ng cDNA template, 0.3 μM of the appropriate primers, and the IQ SYBR Green Supermix 1× (Bio-Rad Laboratories, 1708885). The PCR protocol consisted of 40 amplification cycles (95°C for 30 seconds, 60°C for 45 seconds, and 79°C for 15 seconds) and was performed using an iCycler IQ multicolor Real-Time PCR detection system (Bio-Rad Laboratories), used to determine the threshold cycle (Ct). Melting curves were performed to detect nonspecific amplification products. A standard curve was established for each target gene using 4-fold serial dilutions (from 100 to 0.097 ng) of a cDNA template mix prepared in the same conditions. Each sample was normalized to relative expression level of ribosomal protein L32 (*Rpl32*). Calculation of Ct, standard curve preparation, and quantification of mRNAs in each sample were performed using the “post run data analysis” software provided with the iCycler system (Bio-Rad).

### FFA measurement

Cells in culture (4.10^5^ cells/cm^2^) were analyzed at 13–14 DIV. Total lipids were extracted according to manufacturer guidelines (Free Fatty Acid Quantification Kit, ab65341, Abcam). Briefly, FAs were converted to their CoA derivatives and subsequently oxidized with concomitant generation of color. Octanoate and longer FAs were quantified by colorimetric spectrophotometry (at 570 nm) with detection limit 2 μM FFA in samples. Relative FFAs were quantified based on the protein content.

### Electrophysiology

Cells in culture (8.10^4^ cells/cm^2^) were analyzed at 13–17 DIV. Total synaptic activity was recorded in voltage clamp mode (holding potential –60 mV). The recording bath solution contained 150 mM NaCl, 5.4 mM KCl, 2 mM CaCl_2_, 2.1 mM MgCl_2_, 10 mM HEPES, and 10 mM glucose (pH adjusted to 7.4 with NaOH, osmolarity: 320 mOsm/L at room temperature). Borosilicate glass capillaries were pulled using a P97 horizontal puller (Sutter Instruments) and had a resistance of 4–8 MΩ when filled with the internal solution. The internal pipette solution contained 140 mM KCl, 10 mM EGTA, 10 mM HEPES, 4 mM MgCl_2_, 0.3 mM GTP, and 2 mM ATP-Na_2_ (pH adjusted to 7.2 with KOH, 300 mOsm/L). All recordings were performed at room temperature. Data were acquired using an Axopatch 200B (Axon Instruments), low-pass filtered at 5 kHz, and collected at 10 kHz using a Digidata 1322 A digitizer (Axon Instruments). Once whole-cell configuration was established, liquid junction potential and capacitance transients were compensated. RMP measured in current clamp mode (I = 0) was stable and registered using the built-in voltmeter in the Axopatch 200B. Input and series resistance were monitored during the experiment, and recordings were excluded when any of these parameters changed by more than 10%. Values obtained were annotated in the laboratory protocol notebook. Recordings of total synaptic activity were done for 2 minutes in a gap-free mode using Clampex 10.1, and analysis was performed off-line using Clampfit 10.1 (Axon Instruments) and Excel (Microsoft Corporation).

### Statistics

GraphPad Prism (Version 9.0.0, ref. 121, GraphPad Software Inc) was used for data display and statistical analysis. We did not predetermine sample sizes. The Shapiro-Wilk test was used to test for the normality of data. Parametric testing procedures (Student’s 2-tailed *t* test or 1-way ANOVA) followed by Tukey’s multiple-comparison posttest when many subgroups were compared) were applied for normally distributed data; otherwise, nonparametric tests were used (Mann-Whitney or Kruskal-Wallis test followed by Dunn’s multiple-comparison posttest when many subgroups were compared). Total number of samples (*n*) analyzed in all experimental conditions (number of repeated measurements) is indicated in figure legends. Results were presented as mean ± SEM, and statistical significance was set at *P* < 0.05 (2-tailed tests). In electrophysiology, for non-normally distributed data, Kolmogorov-Smirnov test was selected. Values of *P* < 0.05 were considered statistically significant.

### Study approval

#### Human brain autopsy.

Human brain tissues were obtained from the GIE NeuroCEB (Paris) (https://www.neuroceb.org/en/), Netherlands (Amsterdam) (https://www.brainbank.nl), and ULB LHNN (Brussels) Brain Banks. GIE NeuroCEB’s Brain Bank procedures have been reviewed and accepted by the Ethical Committee “Comité de Protection des Personnes Paris Ile de France VI” and have been declared to the Ministry of Research and Higher Education as requested by the French law. The Netherlands Brain Bank received permission to perform autopsies and to use tissue and medical records from the Ethical Committee of the VU University Medical Center. Tissues from the ULB LHNN Brain Bank were obtained in compliance with and following approval of the Ethical Committee of the Medical School of the Free University of Brussels. An explicit informed consent document had been signed by the patient or by the next of kin, in the name of the patient for autopsy and use of the brain tissue for research purposes.

#### Animals.

All animal procedures used in the study were carried out in accordance with institutional and European guidelines as certified by the local Animal Ethics Committee (Brussels, Belgium). Housing conditions were specified by the Belgian Law of May 29, 2013, regarding the protection of laboratory animals (agreement and project numbers: LA2230419/LA2230652 and 2018/UCL/MD/035).

## Author contributions

NP supervised the project and edited the final version of the manuscript. NP and JNO designed the study. NP, FSO, TL, and FR performed the research. AK, KL, FL, EB, BS, CD, JPB, and PG contributed the new reagents/analytic tools. NP, FSO, and TL analyzed the data. NP and FSO wrote the manuscript in consultation with JNO.

## Supplementary Material

Supplemental data

## Version 1 07/06/2021

In-Press Preview

## Version 2 08/23/2021

Electronic publication
