# Supplementary material for: Regulation of PPARα by APP in Alzheimer disease affects the pharmacological modulation of synaptic activity
Source: JCI Insight. 2021 Aug 23;6(16):e150099. doi: 10.1172/jci.insight.150099 (PMC8410016; doi:10.1172/jci.insight.150099)

Regulation of PPARα by APP in Alzheimer disease impacts the pharmacological modulation of synaptic activity.

Francisco Sáez-Orellana[1†], Thomas Leroy[1], Floriane Ribeiro[1], Anna Kreis[2], Karelle Leroy[3], Fanny Lalloyer[4], Eric Baugé[4], Bart Staels[4], Charles Duyckaerts[5], Jean-Pierre Brion[3], Philippe Gailly[2], Jean-Noël Octave[1], and Nathalie Pierrot[1,†,*].

[1] Université catholique de Louvain, Institute of Neuroscience, Alzheimer Dementia group, B-1200 Brussels, Belgium.

[2] Université catholique de Louvain, Institute of Neuroscience, Laboratory of Cell Physiology, B-1200 Brussels, Belgium.

[3] Université libre de Bruxelles, Laboratory of Histology and Neuropathology, 1070 Brussels, Belgium.

[4] Université de Lille EGID, Inserm, CHU Lille, Institut Pasteur de Lille, U1011, F-59000 Lille, France.

[5] Sorbonne Université, Hôpital de la Pitié-Salpêtrière, Paris Brain Institute (ICM), CNRS UMR7225, INSERM U1127, 75013 Paris, France.

† These authors contributed equally to this work.

* Correspondence: Nathalie Pierrot, Université catholique de Louvain, Institute of Neuroscience, Alzheimer Dementia, avenue Mounier 53, SSS/IONS/CEMO-Bte B1.53.03, B-1200, Brussels, Belgium. Office: +32 2 764 93 34, E-mail: nathalie.pierrot@uclouvain.be

Thomas Leroy's present affiliation: VIB Center for Brain and Disease Research & KU Leuven, Department of Neurosciences, Gasthuisberg, B-3000 Leuven, Belgium

Conflict of interest statement:

The authors have declared that no conflict of interest exists.

Supplemental Material content:

- Supplemental Methods
- Supplemental References
- Supplemental Tables
- Supplemental Figures

I- Supplemental Methods

Cell cultures. Cortical cultures were prepared from embryonic day 17 (E17) to E18 Wistar rats or P0-P1 pups from *Ppara*$^{-/-}$ and wild-type (WT) mice from the same genetic background of either sex. Pregnant rats and mice were euthanized with CO_2. Cortices were isolated as previously described (1, 2). Cortical cells were plated in culture dishes (4.10^5 cells/cm^2) pre-treated with 10 µg/ml poly-L-lysine (Sigma-Aldrich) in phosphate buffered saline (PBS) and cultured for 13-14 days *in vitro* (DIV) in Neurobasal medium supplemented with 2% (v/v) B-27 medium and 0.5 mM L-glutamine without antibiotic solution prior to analyses. The cultures were maintained at 37°C under a 5% CO_2 atmosphere and half of the medium was renewed every 2-3 days.

Recombinant viruses and cell transduction. At 6 DIV, cells were infected at a multiplicity of infection (MOI) of 10 in a minimal volume of culture medium for 4 h with adenoviruses and up to analysis with lentiviruses (see below). Infection medium was replaced by fresh culture medium every two days.

Adenoviruses. Recombinant adenoviruses encoding wild-type human APP695 (AdhAPP) or human recombinant Green Fluorescent Protein (AdhrGFP) (1.10^{10} PFU mL, Vector Biolabs, #1060) were used. AdhAPP was prepared by cloning the pENTCMV shuttle vector containing the cDNA encoding human APP695 into the pAd-REP and was then amplified and purified (1.10^{12} viral particles / mL, GeneCust).

Construction of lentiviral vectors. Lentiviruses encoding short hairpin RNA (shRNA) construct designed to target mouse/rat APP transcript (shAPP) and a scrambled shRNA encoding GFP (shScra-GFP) used as a negative control were produced. pLKO.1 vectors shAPP were selected using MISSION® shRNA Bacterial Glycerol Stock (Sigma-Aldrich, #SHCLNG-

NM_007471; TRCN0000054874 Clone ID: NM_007471.2-2185s1c1 and TRCN0000054876 Clone ID: NM_007471.2-1583s1c1) and used for construction of recombinant lentiviruses, as previously described (3). Briefly, HEK293T/17 cells (6.10^3 cells / cm^2) (ATCC®, Manassas, VA, USA, catalog no. CRL-11268™) were cultured in DMEM Nutrient Mix F12 supplemented with 10% Foetal Calf Serum and 0,6% Penicillin-Streptomycin (10000 U / mL) for 24 h prior transfection with Mirus TransIT-293 (Sopachem, #MIR 2700) of the pKLO.1 target plasmid (6 µg) together with 4.5 µg pCMV delta R8.2 and 1.8 µgpMD2.G lentiviral packaging plasmids (gift from Didier Trono (Addgene plasmids #12263 and #12259; http://n2t.net/addgene:12263 and 12259; RRID:Addgene_12263 and _12259). Supernatants containing the lentiviruses were harvested 48 h after transfection. Lentiviruses were concentrated and purified with the Lenti-X ™ Concentrator kit (Clontech Laboratories, #PT4421-2) according to the manufacturer's instructions and 20µl of the concentrated lentiviral solution was added to cortical cultures.

Immunoblotting analysis

Cells in culture were washed, scraped off in PBS and centrifuged for 2 min at 16 000 g. Pellets were sonicated in lysis buffer (125 mM Tris (pH 6.8), 20% glycerol, and 4% sodium dodecyl sulfate) with cOmplete Protease Inhibitor Cocktail (Roche, #11697498001). For brain proteins extraction, samples were homogenized in RIPA buffer (1% NP40, 0.5% deoxycholic acid, 0.1% SDS, 150 mM NaCl, 1 mM EDTA, 50 mM Tris, pH 7.4) containing proteases and phosphatases inhibitors cocktail (Roche, #04906837001). The samples were clarified by centrifugation at 20 000g and the protein concentration was determined using a Bicinchoninic Acid Assay (BCA) kit. Samples were heated for 10 min at 70°C in loading buffer (lysis buffer containing 10 % 2-mercaptoethanol and 0.004 % bromophenol blue). Cell and brain lysates (40 µg of proteins) were analyzed by Western blotting using 4-12 % NupageTM bis-Tris gels. Nitrocellulose membranes were incubated overnight at 4 °C with primary antibodies as indicated in

Supplemental Table 4. Blots were incubated with HRP peroxidase-conjugated secondary antibodies (1:10000), revealed by ECL (Amersham Pharmacia, #ORT2655-2755) and quantified using the Quantity OneTM software (Bio-Rad Laboratories). α-tubulin was used as internal standard to normalize protein load in gel.

Semi-quantitative RT-PCR. Total RNA was isolated from primary cultures of mouse cortical cells prepared from wild type and *Ppara* deficient mice using TriPure Isolation Reagent and 1 μg of total RNA was reverse-transcribed (see above, RNA extraction section). Semi-quantitative RT-PCR was carried out after treating total RNA with DNAse to remove any contaminating genomic DNA. The resulting cDNA was appropriately diluted and amplified using TaqDNA polymerase. Glyceraldehyde-3-phosphate dehydrogenase (*Gapdh*) was used to ascertain that an equivalent amount of cDNA was synthesized from different samples. Primers (Sigma-Aldrich) used are described in Supplemental Table 5. Amplified products were electrophoresed on a 2% agarose gel and visualized by Midori Green Advance DNA staining (Nippon Genetics Europe, #MG04) with an electrophoresis gel imaging system (Bio-Rad GelDoc 2000).

II- Supplemental References

1. Pierrot N, Tyteca D, D'auria L, Dewachter I, Gailly P, Hendrickx A, et al. Amyloid precursor protein controls cholesterol turnover needed for neuronal activity. *EMBO Molecular Medicine.* 2013;5(4):608-25.
2. Seibenhener ML, and Wooten MW. Isolation and culture of hippocampal neurons from prenatal mice. *J Vis Exp.* 2012(65).
3. Salmon P, and Trono D. Production and titration of lentiviral vectors. *Curr Protoc Hum Genet.* 2007;Chapter 12:Unit.

III- Supplemental Tables

	Case number	Age at death (years)	Sex	ApoE genotype	Post-mortem interval (h)	Braak stage	CERAD Plaque score
Control subjects	1	79	M	ε3/ε3	6.5	I	A
	2	79	M	ε3/ε3	6	II	A
	3	78	F	NA	7	I	A
	4	78	F	ε3/ε3	4.5	II	A
	5	89	F	ε2/ε4	35	0-I	A
	6	72	M	ε3/ε3	24	0-I	A
	7	71	F	ε2/ε3	7	0-I	A
	8	77	F	ε3ε/4	48	0-I	A
LOAD	1	77	M	NA	6.5	VI	C
	2	74	M	ε4/ε3	7.5	VI	C
	3	72	M	ε4/ε2	5.3	VI	C
	4	72	F	ε4/ε4	6.5	VI	C
	5	73	F	ε4/ε4	22	V-VI	C
	6	70	F	ε3/ε3	21	V-VI	C
	7	91	F	ε3/ε4	5.5	V-VI	C
	8	84	M	ε3/ε3	7	V-VI	C
	9	89	F	ε2/ε4	7	V-VI	C

	Post-mortem interval (h) Mean ± SEM	Age at death (years) Mean ± SEM	Sex F/M
Control subjects	17.2 ± 5.8	77.8 ± 1.9	5/3
LOAD	9.8 ± 2.2[a]	78 ± 2.6[b]	5/4

Supplemental Table 1. **Clinical information on sporadic Alzheimer disease patients**. Means of post-mortem interval ([a]) and age ([b]) were not significantly different between control subjects (n=8) and late-onset Alzheimer disease cases (LOAD, n=9) (P = 0.5858 and P = 0.9707, Mann-Whitney and Student's *t* tests, respectively). The neuropathological staging of AD patients is determined according to the Braak and Braak staging and semi-quantitative measure of neuritic plaque density has been estimated as recommended by the Consortium to Establish a Registry for Alzheimer's disease (CERAD). ApoE, apolipoprotein E; NA, not-available; M, male; F, female.

	Case number	Age at death (years)	Sex	ApoE genotype	Post-mortem interval (h)	Braak stage	CERAD Plaque score
**Control subjects**	1	58	M	NA	5.5	III	NA
	2	69	M	NA	6	0	NA
***APP*dup**	3	54	M	NA	NA	VI	NA
	4	59	M	NA	5.5	V	C

	**Post-mortem interval** (h) Mean ± SEM	**Age at death** (years) Mean ± SEM	**Sex F/M**
**Control subjects**	5.7 ± 0.2	63.5 ± 5.5	0/2
***APP*dup**	5.5 ± 0.0[a]	56.5 ± 2.5[b]	0/2

Supplemental Table 2. **Clinical information on patients with microduplication of the *APP* locus**. Means of post-mortem interval ([a]) and age ([b]) were not significantly different between control subjects (n=2) and early-onset Alzheimer disease rare cases with an *APP* duplication locus (*APP*dup, n=2) (P = 0.6667 and P = 0.3662 Student's t test,). The neuropathological staging of AD patients is determined according to the Braak and Braak staging and semi-quantitative measure of neuritic plaque density has been estimated as recommended by the Consortium to Establish a Registry for Alzheimer's disease (CERAD). ApoE, apolipoprotein E; NA, not-available; M, male; F, female.

Mouse strain	Primers name	Sequence 5' to 3'
Ppara$^{-/-}$ (JAX stock #008154)	oIMR8075 (Common)	GAGAAGTTGCAGGAGGGGATTGTG
	oIMR8076 (Wild type Reverse)	CCCATTTCGGTAGCAGGTAGTCTT
	oIMR8077 (Mutant Reverse)	GCAATCCATCTTGTTCAATGG C
APPWt line I5 (*hAPP*) (JAX stock #004662)	oIMR2044 Transgene Forward	GGTGAGTTTGTAAGTGATGCC
	oIMR2045 Transgene Reverse	TCTTCTTCTTCCACCTCAGC
	oIMR8744 Internal Positive Control Forward	CAAATGTTGCTTGTCTGGTG
	oIMR8745 Internal Positive Control Reverse	GTCAGTCGAGTGCACAGTTT

Supplemental Table 3. Primers used for genotyping.

Protein	Antibody (Clone)	Epitope / Immunogen	Working dilution	Source (catalog)
Amyloid β (APP)	mouse monoclonal (WO-2)	Synthetic peptide from human Amyloid-β (aa 4-10)	1 : 2000	Millipore (MABN10)
APP, C-Terminal	rabbit polyclonal	Synthetic peptide from C-terminal of human APP_{695} (aa 676-695)	1 : 4000	Sigma-Aldrich (A8717)
α-Tubulin	mouse monoclonal (B-5-1-2)	Epitope located at the C-terminal end of the α-tubulin isoform	1 : 4000	Sigma-Aldrich (T6074)
GFP	mouse monoclonal (clone 7.1 and 13.1)	Originally isolated from the jellyfish *Aequorea victoria*	1 : 2000	Roche (11814460001)

Supplemental Table 4. Antibodies, sources, applications and working dilutions used in this study.

Gene	Accession number (RefSeq)	Primers 5' to 3'<br>F, Forward; R ,Reverse
***Ppara* mouse**	**NM_011144**	F- AAACTTGGACTTGAACGACC<br>R- GCATCCCGTCTTTGTTCA
***Gapdh* mouse**	**NM_008084**	F- CATGGCCTTCCGTGTTCCTA<br>R- GCGGCACGTCAGATCCA
***ACOX1* human**	**NM_004035.7**	F- GCGTTATGAGGTGGCTG<br>R- TCAGCGATGCCAAACTC
***CPT1A* human**	**NM_001876.4**	F- CAAGATGAGTCGTGCCA<br>R- CGAGGCAGCGATGTCT
***PDK4* human**	**NM_002612.4**	F- GGAAACCCAAGCCACATT<br>R- CACAGAGCATCCTTGAACACT
***RPL32* human**	**NM_000994.4**	F- CGTAACTGGCGGAAAC<br>R- TGGCCCTTGAATCTTCTA
***Acox1* mouse/rat**	**NM_001271898.1**	F- GCTGGGCTGAAGGCTTT<br>R- GCTGTGAGAATAGCCGTG
***Cp1a* rat**	**NM_001031847.2**	F- CGCAAAGATCAGTCGGA<br>R- ACGCCGCTCACAATG
***Cpt1a* mouse**	**NM_013495.2**	F- TGGCTTATCGTGGTGGT<br>R- GTGTCTAGGGTCCGATT
***Pdk4* mouse/rat**	**NM_013743.2 mouse**<br>**NM_053551.1 rat**	F- ACACGCTGGTCAAAGTTC<br>R- TGAGCATCCGAGTAGAAAT
***Rpl32* rat**	**NM_013226.2**	F- CGAAACTGGCGGAAAC<br>R- TGGCCCTTGAATCTTCTC
***Rpl32* mouse**	**NM_172086.2**	F- CGAAACTGGCGGAAAC<br>R- TGGCCCTTGAACCTTCTC

Supplemental Table 5. Primers used for RT-PCR and real-time PCR analyses.

IV- Supplemental Figures

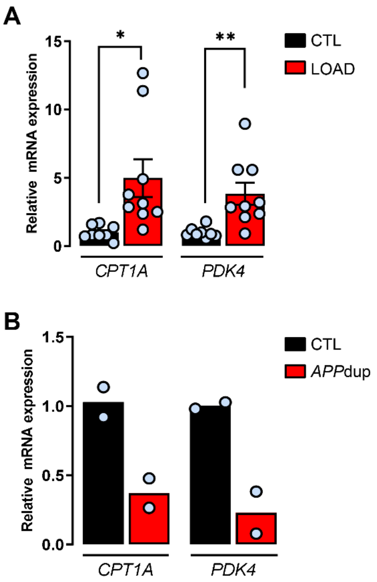

Supplemental Figure 1. PPARα downstream target genes expression in brains from patients with Alzheimer disease. Frontal cortex of postmortem human brain tissues from late-onset (LOAD, n = 9) and early-onset Alzheimer disease cases with an *APP* duplication locus (*APP*dup, n = 2) and respective control subjects (CTL in LOAD and *APP*dup cases, n = 8 and 2, respectively) were analyzed. (**A** and **B**) Quantitative real time PCR analyses for *CPT1A* and *PDK4* mRNA levels. Results were normalized to *ACTB* mRNA and relative differences are expressed according to respective CTL as mean ± SEM (LOAD: *CPT1A* mRNA, $P = 0.016$; *PDK4* mRNA, $P = 0.017$; Student's t-test), $*P < 0.05$, $**P < 0.01$.

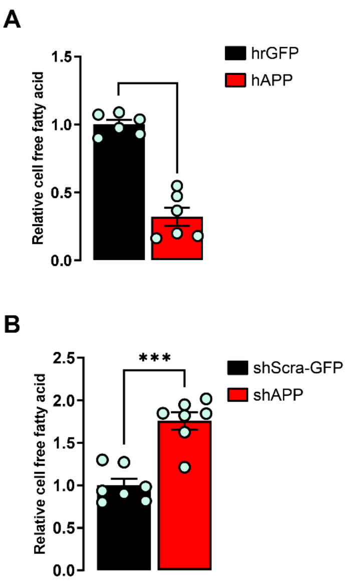

Supplemental Figure 2. Free fatty acid content in cortical cultured cells. Primary cultures of rat cortical cells were infected with adenoviruses encoding human recombinant GFP (hrGFP) or APP (hAPP) (**A**, n = 6) or with lentiviruses encoding a shRNA targeting endogenous APP (shAPP) or a scrambled shRNA encoding GFP (shScra-GFP) (**B**, n = 7). At 13-14 DIV, cell free fatty acid content was measured in 3 independent experiments. Relative differences are expressed according to respective control as mean ± SEM; ***$P < 0.0001$; Student's t-test.

D Full unedited gel for Figure 1

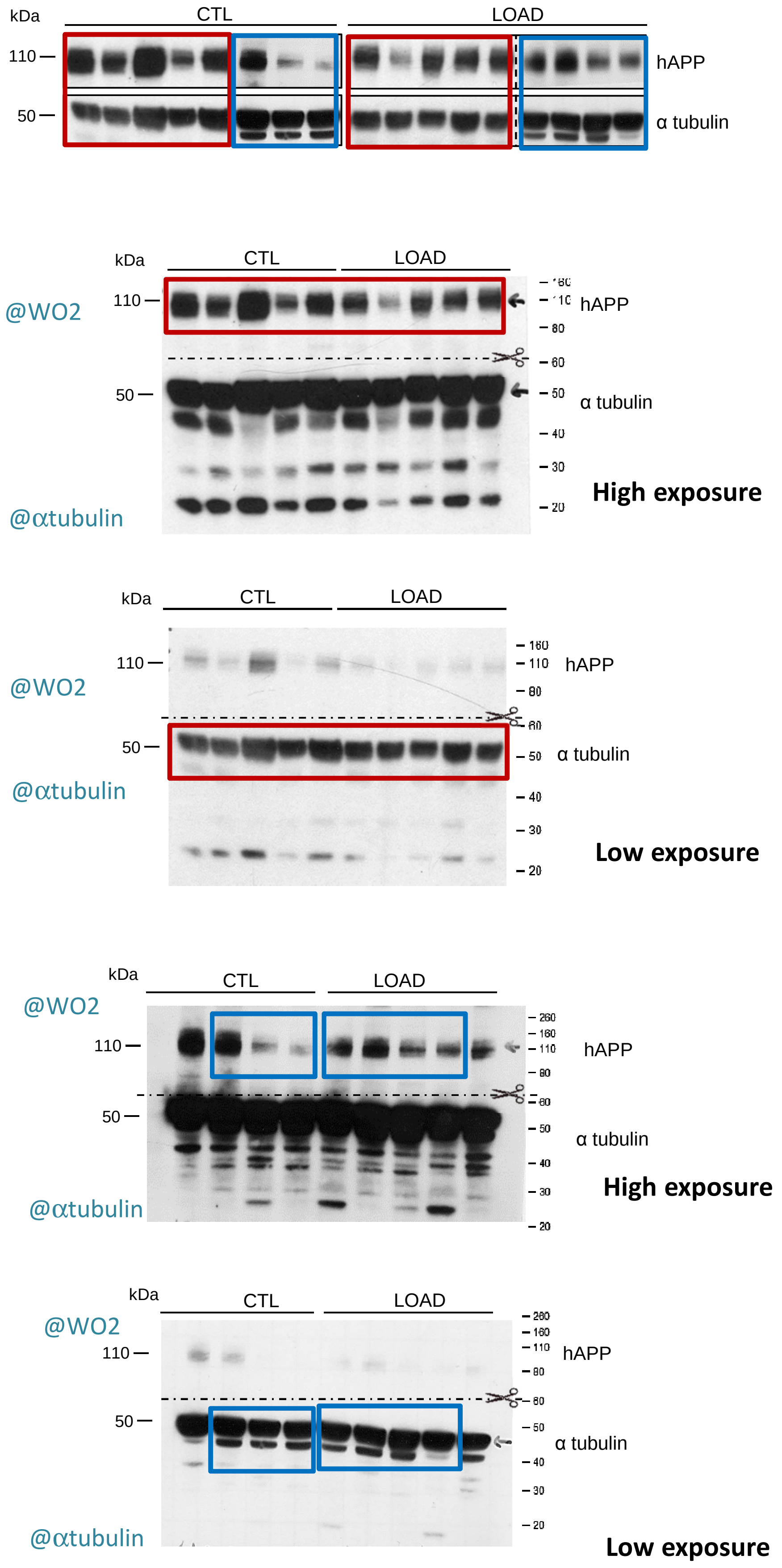

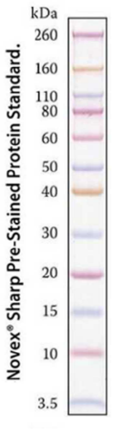

Full unedited gel for Figure 1

J

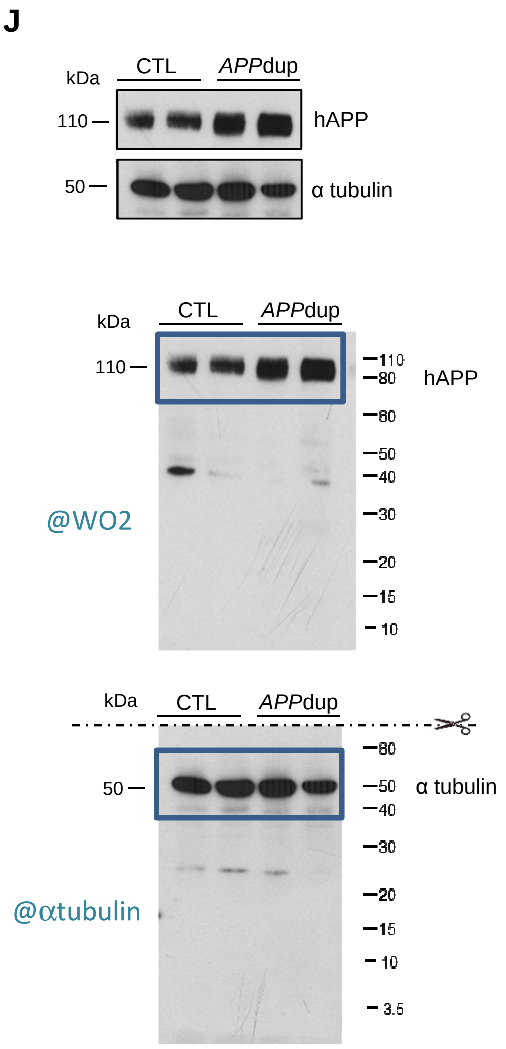

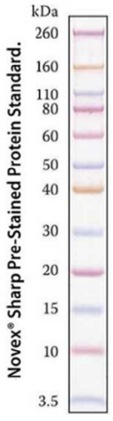

A

Full unedited gel for Figure 2

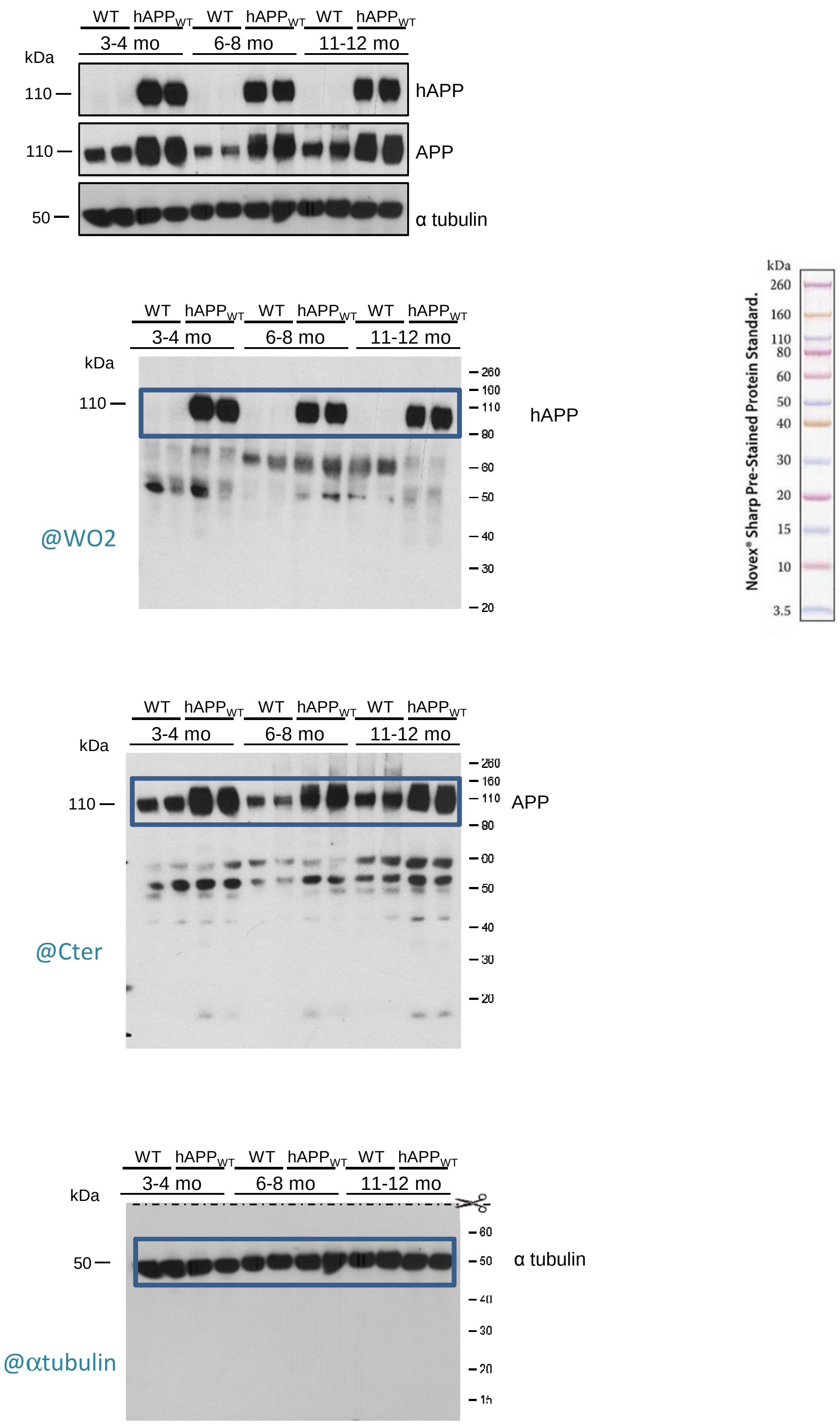

Full unedited gel for Figure 3

A

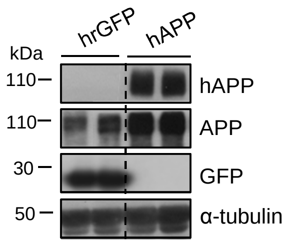

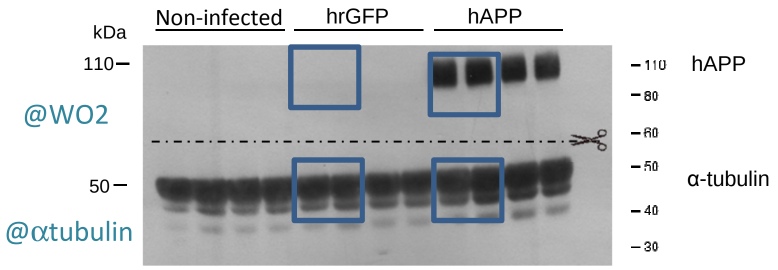

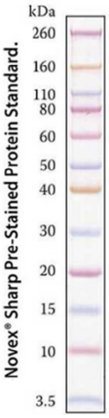

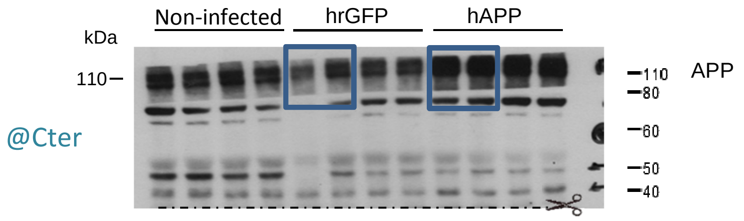

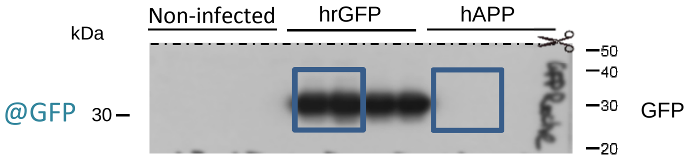

Full unedited gel for Figure 4

A

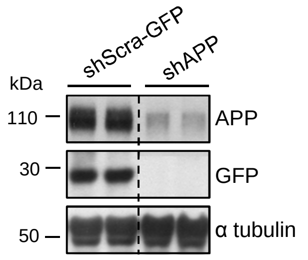

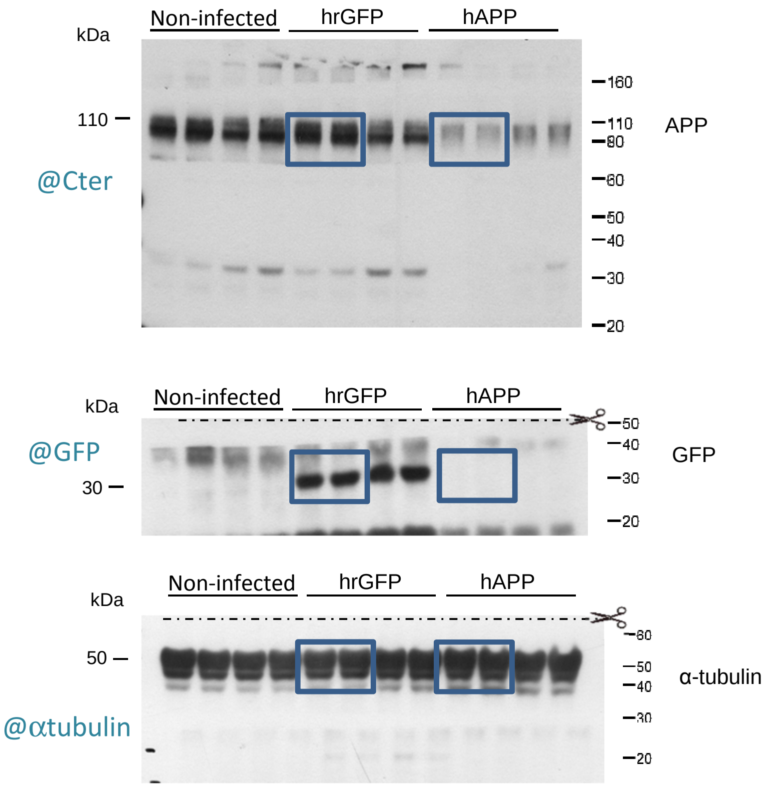

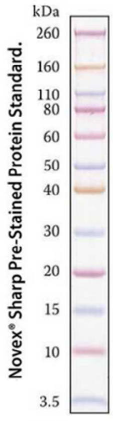

Full unedited gel for Figure 5

A

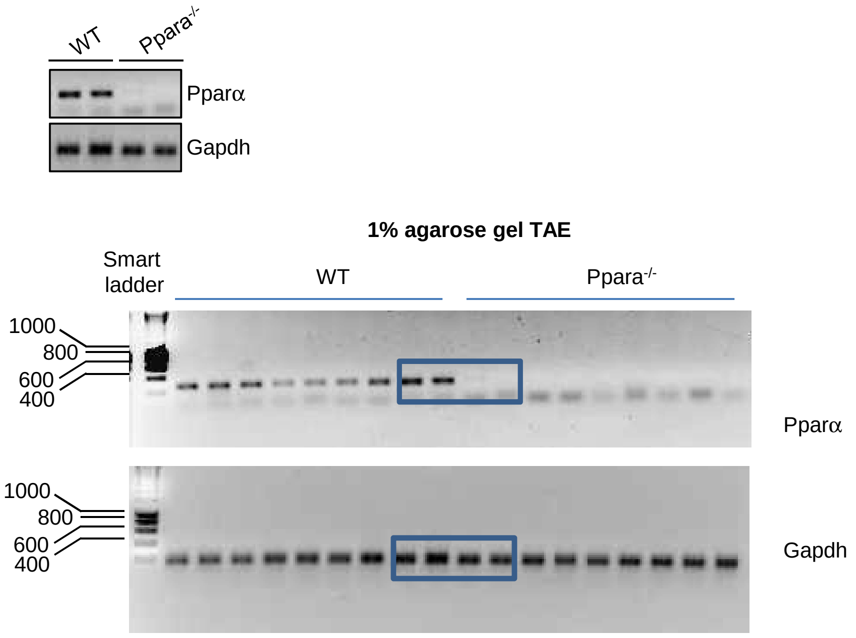

Full unedited gel for Figure 5

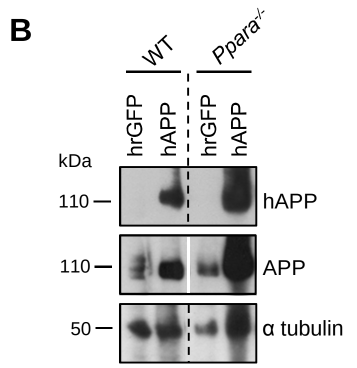

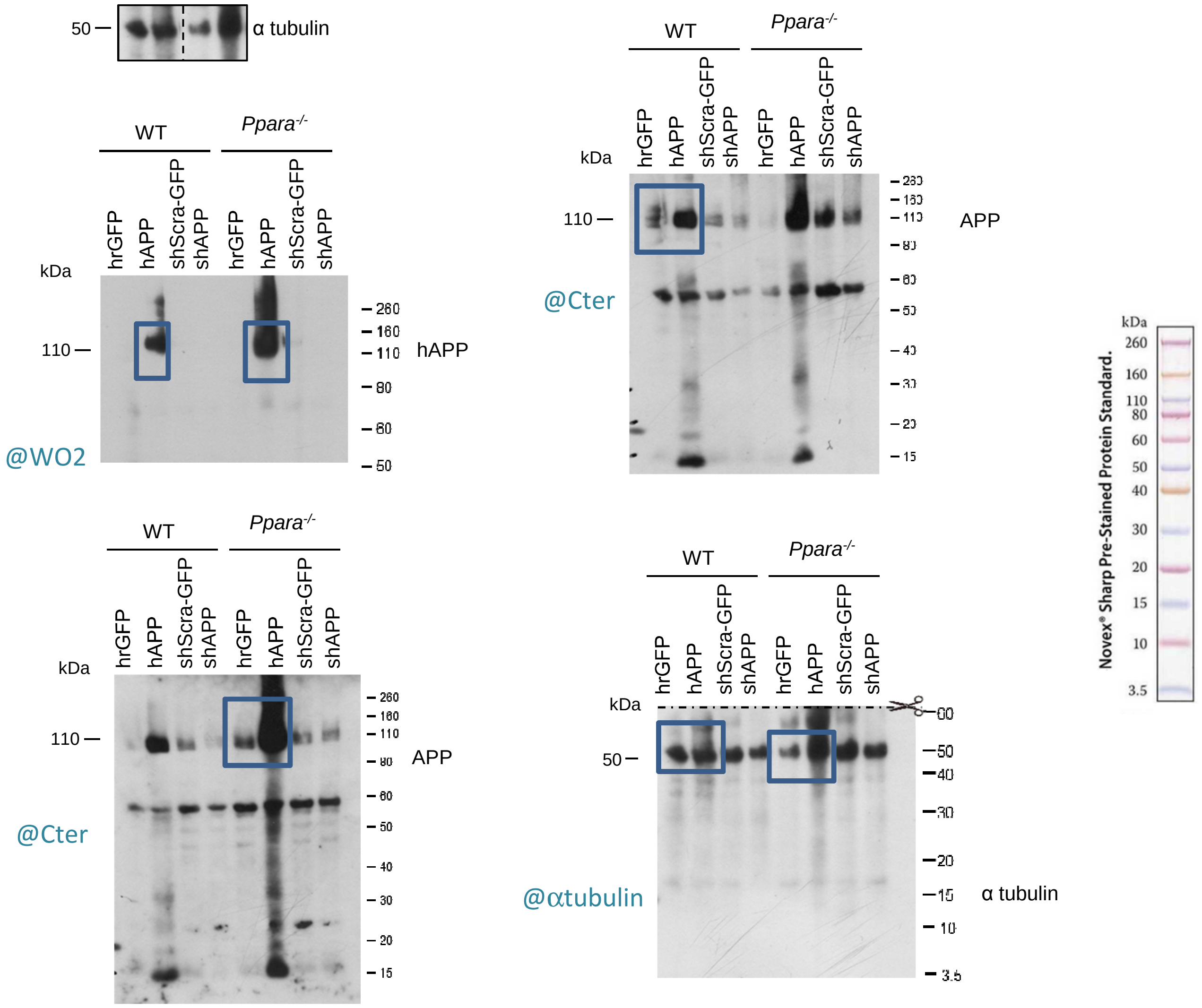

Full unedited gel for Figure 5

C

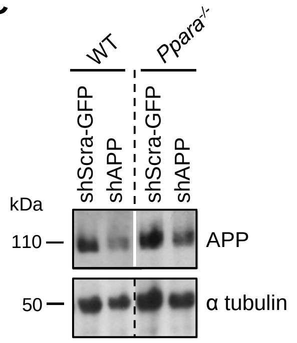

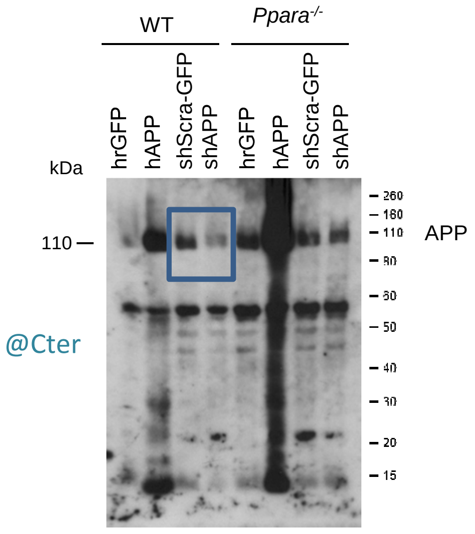

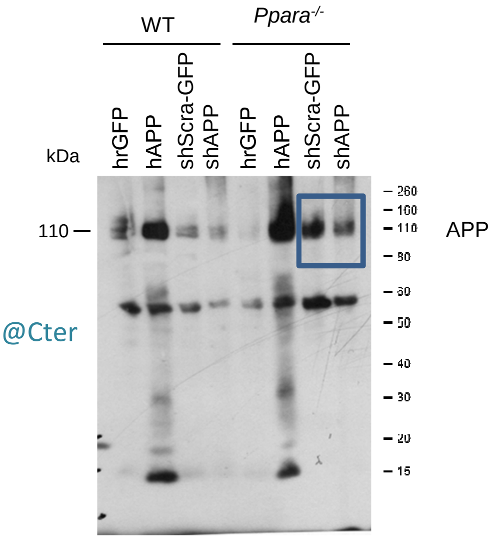

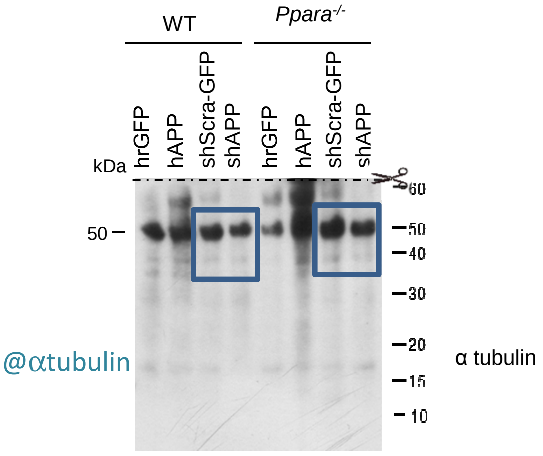

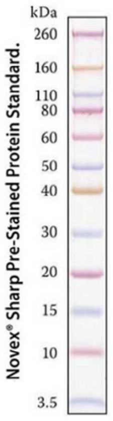

Supplement: Supplemental data [file jciinsight-6-150099-s055.pdf]
